# Ultra‐Fast Portable and Wearable Sensing Design for Continuous and Wide‐Spectrum Molecular Analysis and Diagnostics

**DOI:** 10.1002/advs.202203693

**Published:** 2022-10-20

**Authors:** Arnab Maity, Yana Milyutin, Vivian Darsa Maidantchik, Yael Hershkovitz Pollak, Yoav Broza, Rawan Omar, Youbin Zheng, Walaa Saliba, Tan‐Phat Huynh, Hossam Haick

**Affiliations:** ^1^ Department of Chemical Engineering and Russell Berrie Nanotechnology Institute Technion – Israel Institute of Technology Haifa 3200003 Israel; ^2^ Laboratory of Molecular Science and Engineering Faculty of Science and Engineering Abo Akademi University Henrikinkatu 2 Turku FI‐20500 Finland

**Keywords:** wearable devices, spectromerty, hierarchical electronics, sensors, molecular analysis, breast cancer, chiral molecules, volatile organic compound

## Abstract

The design and characterization of spatiotemporal nano‐/micro‐structural arrangement that enable real‐time and wide‐spectrum molecular analysis is reported and demonestrated in new horizons of biomedical applications, such as wearable‐spectrometry, ultra‐fast and onsite biopsy‐decision‐making for intraoperative surgical oncology, chiral‐drug identification, etc. The spatiotemporal sesning arrangement is achieved by scalable, binder‐free, functionalized hybrid spin‐sensitive (<↑| or <↓|) graphene‐ink printed sensing layers on free‐standing films made of porous, fibrous, and naturally helical cellulose networks in hierarchically stacked geometrical configuration (HSGC). The HSGC operates according to a time‐space‐resolved architecture that modulate the mass‐transfer rate for separation, eluation and detection of each individual compound within a mixture of the like, hereby providing a mass spectrogram. The HSGC could be used for a wide range of applictions, including fast and real‐time spectrogram generator of volatile organic compounds during liquid‐biopsy, without the need of any immunochemistry‐staining and complex power‐hungry cryogenic machines; and wearable spectrometry that provide spectral signature of molecular profiles emiited from skin in the course of various dietry conditions.

## Graphical abstract

The current article reports on the development of spatiotemporal nano‐/micro‐structural arrangement that enable a wide spectrum of chemicals and complex mixtures to be separated and detected simultaneously when used as wearable‐spectrometry or as ultra‐fast and onsite biopsy‐decision‐making tool for intraoperative surgical oncology. The reported arrangement exhibits rapid discrimination of cancer from healthy tissues as well as spectral signature of volatile organic compounds emanated directly from tumor/skin.

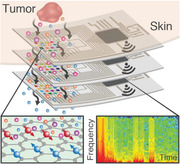

## 1 Introduction

Despite the steady and gradual advancements in recent decades, contemporary medicine still suffers from impactful shortcomings in successful disease diagnosis and treatment, which relies not only on its detection capacity but also on timing.^[^ ^1^, ^2^, ^3^ ^]^ A promising approach to overcome these shortcomings is by shifting the hospital‐centered healthcare system to on‐site and real‐time detection and monitoring of an individual's health, feeding relevant information back to the users and/or medical professionals, and sounding an alarm when an adverse condition is encountered.^[^ ^4^ ^]^ Recent insights in this endeavor have resulted in a rapid development of various wearable devices monitoring different physical parameters, such as pressure/strain, body vital signs (e.g., heartbeat rate, respiration rate, and temperature) as well as biomarkers that can be found in various body fluids, for example, blood, saliva, sweat, tears, skin‐odor, and breath.^[^ ^5^, ^6^ ^]^ Nevertheless, most of these devices are limited in the scope of their detection capabilities and/or the type of molecular details and diagnostics they can provide.

Wearable devices that rely on selective/lock‐and‐key sensors can be tuned to enable targeted identification, viz. detection of specific and well‐defined targeted molecules in the presence of interfering species or background.^[^ ^7^, ^8^, ^9^ ^]^ This group of sensors requires laborious identification of the targeted molecule in the presence of interfering background as well as the synthesis of a highly selective nanomaterial for each molecule of interest. Additional limitation stems from the need to synthesize separate, highly selective materials for each targeted molecule to be detected, mainly when the targets are non‐polar. Wearable devices that rely on cross‐reactive sensors in conjugation with pattern recognition methods are more suitable for rapid detection of chemical compounds and their mixtures.^[^ ^10^ ^]^ Nevertheless, this approach provides a superimposed signal(s), that is, a weighted sum of the responses of the individual components or the overall collective fingerprint of the sample, from all compounds in the sample.^[^ ^11^ ^]^ Additionally, the countless possible combinations of components in a mixture, which need to be tested and trained for a reliable prediction in real sample analysis, complicates the prediction of each individual compound in real sample analysis and decreases its reliability.

The current article reports on a flexible biocompatible spatiotemporal sensing architecture that offers ultra‐fast and wide‐spectrum molecular analysis, suspect screening, and discovery analysis based on both target and non‐target approaches for on‐site and on‐body biomedical applications. The article starts by introducing the concept and fabrication of the spatiotemporal sensing architecture as well as the characterization of the structure–property relationship. This part is followed by discussing the mechanism behind the powerful detection and classification capabilities of this sensing architecture, using theoretical and calculation modeling. Then, the reported architecture is applied and examined for rapid discrimination of breast cancer tissues as well as for wide‐spectrum skin‐based molecular analysis of health and dietary conditions. The excellent spin‐sensitivity and magnetic influence of chiral molecules are also demonstrated for rapid chiral‐drug detection and chiral‐spintronics application.

## 2 Results and Discussion

### 2.1 Characterization of Hybrid DrGO and FDrGO

**Figure 1:**
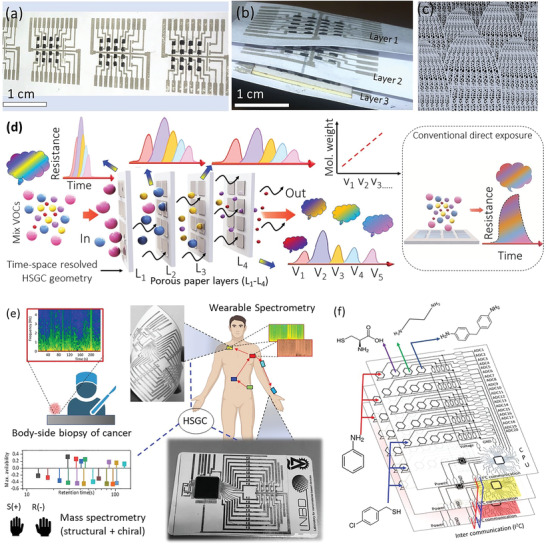
Nature‐inspired HSGC spectrometry. a,b) HSGC planar and folded structure printed with FDrGO sensor pixel array (4 × 5 matrix) between silver nanowire‐based multijunction printed electrodes on porous paper substrate inspired from butterfly wings (c), see also Figure S1a,b, (Supporting Information); d) Schematic model of HSGC‐based layered exposure to show Spatio‐temporal splitting and its comparison with direct exposure to sensor (shown in dotted inset). e) Biomedical application of HSGC structure simultaneous structural/chiral recognition, real time rapid biopsy of a cancerous tumor, wearable spectrometry on human skin. A photograph of flexible circuit embedded with a microprocessor‐based computing unit is also shown in the inset; f) circuit schematic for communicating (inter‐integrated controller (I2C) protocol) all printed pixels from printed layers. The typical ligands are mentioned near each pixel (see a detailed list of ligands in Table S1 Supporting Information).

**Figure** **1** shows the design and examination of a spatiotemporal nano‐/micro‐structural arrangement of miniaturized sensors with the capabilities of spectrometers for identifying complex structural and chiral molecules using hierarchically stacked geometrical configuration (HSGC) architecture. This arrangement (Figure 1a,b) is very similar to the butterfly's layer‐by‐layer micro‐wing structure and its porous nature (Figure 1c; Figure S1a,b, Supporting Information). The fabrication of the HSGCs starts with a binder‐free inject‐printing of an array (2D matrix (4 × 5)) of dopamine‐reduced graphene oxide (DrGO) sensors functionalized with a variety of chemical ligands on a layer made of porous cellulose paper (see Figure 1a,b). Details of the hybrid DrGO synthesis and printing technique are provided in the Experimental Section and Section S1, Table S1, and Figures S1–S3, Supporting Information. A comparison of the binder‐ and surfuctant‐free printing of the 2D materials with state‐of‐the‐art approaches is provided in Table S2, Supporting Information. The binder‐ and surfactant‐free strategy does not need any post‐printing annealing and therefore is highly suitable for paper‐based sustainable electronics. A real‐time video of the binder‐free printing process is provided in Video S1, Supporting Information. The choice of using DrGO and its derivatives as sensor‐ink, compared to other emerging materials (such as MXene and pnictogens), is because of the easy fabrication/printing procedure, low cost, scalability, and good stability. The almost negligible cytotoxicity that is highly sought after for large scale industrial process engineering and in various wearable applications for medical electronics is another reason for this choice. Silver nanowire‐based interdigitated conducting electrodes are printed to connect all pixels in the matrix (see photograph of printed electrode on paper in Figure 1a and synthesis procedure of Ag nanowire in Experimental Section and Section S2, Figure S4, Supporting Information); the typical resistance variation for various print pass is shown in Figure S1c, Supporting Information. A typical bending experiment shows that the resistance of a printed network changes only minimally during full bending or folding (Section S3, Figure S5, Supporting Information). Then, a multiplicity of these layers is stacked together, in a way that the FDrGO sensors face the bottom side of the previous cellulose layer in the stack. Upon exposure to a mixture of chemicals, each layer separates substances based on differential adsorption of compounds to the adsorbent as the compounds move through the layer at different diffusion rates as per Graham's law, which states that the diffusion rate of a molecule is inversely proportional to the square root of molar mass. Therefore, in a mixture, the rate of diffusion of gas molecules of lower mass would be faster than the heavier molar mass‐based molecules allowing their separation in fractions. Then, the compounds with the lower adsorption and/or affinity to the stationary phase travel faster than those having greater adsorption and/or affinity with the stationary phase (see schematic in Figure 1d). The compounds that move faster, reach, and interact are sensed by the array of FDrGO sensors first in each of the HSGC's layers. This process induces a nonlinear resistance profile with multiple peaks at different times from sensors exposed in each HSGC layer (LE) (see Figure 1d). This architecture could be thought of as chromatography columns (the porous cellulose paper) and mass spectrometer (array of sensors) that are connected in a series, assuring high‐resolution separation, elution, and detection of chemicals from a mixture of compounds. Using this framework, we demonstrated the potential to form an all inkjet‐printed heterostructure for mass scale printing and a microprocessor‐based computational unit (Mega 2560 IC) to interface sensor pixels on a credit card‐sized printed flexible paper inset picture (Figure 1e) with the relevant circuit of a typical single layer of the HSGC arrangement. Multi‐layer arrangement with typical ligand names is shown in Figure 1f.

**Figure 2:**
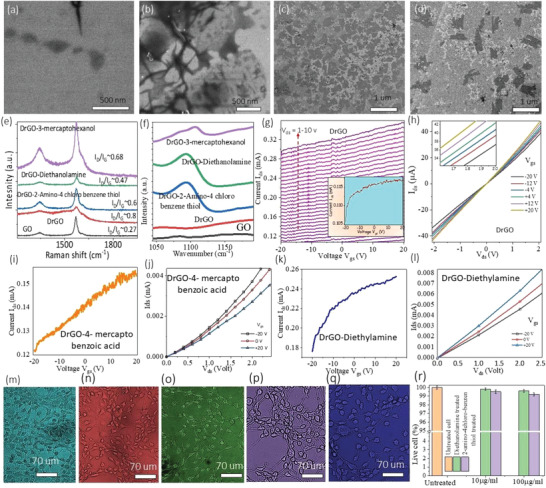
Characterization of GO, DrGO, and FDrGO structures. a–d), Typical SEM image of microstructures for GO, DrGO, thiol‐DrGO, and amine‐DrGO respectively. e–l) Raman spectroscopy, FTIR, FET, and I‐V characteristics for GO, DrGO, and various FDrGO samples with calculated I_D_/I_G_ ratio as noted in the figure. Cytotoxic assessment of m) untreated human lung epithelial cells (BEAS‐2B, ATCC, and CRL‐9609) to treat with n,o) typical amine (Diethalolamine) and p,q) thiol (2‐amino‐4‐chloro‐benzene thiol) terminated FDrGO samples for 10–100 µg mL^−1^ respectively. r) The live cell% after 24 h treatment is compared with untreated cells.

**Figure** **2**a–l shows representative results of scanning electronic microscopy (SEM), Raman, Fourier transformed infrared spectroscopy (FTIR), field effect transistor (FET) and current–voltage (*I*–*V*) data for graphene oxide (GO), DrGO, and thiol‐/amine‐/chiral/achiral‐FDrGO to identify morphological, structural, functionalization status and basic electronic quality of materials (see details in Section S4, Supporting Information). Figure S6 of the Supporting Information shows Nuclear Magnetic Resonance (NMR) spectrum of FDrGO samples for estimating the load of the surface chemistry. Biocompatibility and cytotoxicity studies of synthesized FDrGO materials were carried out on human lung epithelial cells (BEAS‐2B, ATCC, and CRL‐9609) (Figure 2m) with various biochemical ligands modified with FDrGO inks (Figure 2n‐q) at 10 and 100 µg mL^−1^ dosages, respectively (24 h treatment time). Figure 2r relates the percentage of living cells to various FDrGO ink concentrations; the dosage was followed by dye treatment with Hoechst 33 342 (≈0.2 µg mL^−1^) for imaging. The results indicated that the designed HSGC structures were biocompatible and non‐toxic (see more in Section 4.6, Supporting Information). Section S3 and N‐type semiconducting properties (FET) of DrGO/FDrGOs suggest integration of N_2_ adatom at graphitic plane during GO‐reduction with polydopamine (see quantum mechanical density functional theory (DFT) analysis in chiral recognition experimental analysis (section 2.3.1). The FET measurement platform is schematically shown in Figure S7, Supporting Information.

### 2.2 Layer‐Dependent Sensing using HSGC Architecture

**Figure 3:**
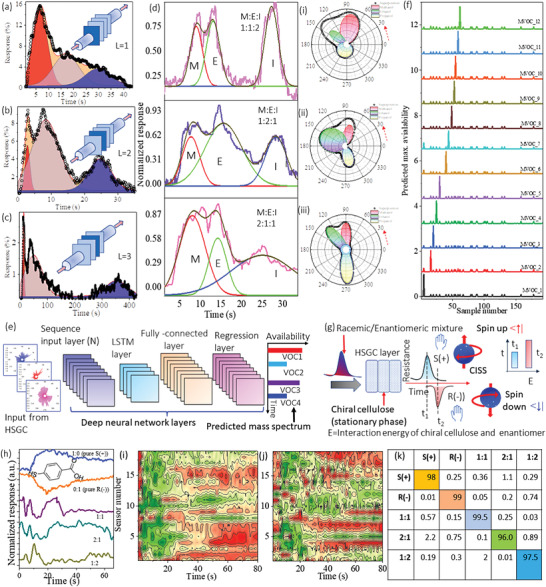
Prismatic gas dispersion properties of HSGC. a–c) HSGC‐derived chromatographic separation of a typical VOC mixture, methanol:ethanol: isopropanol (M:E:I = 1:1:1), at layers 1, 2, and 3 respectively. d) Using layer 2 HSGC (optimized), the M:E:I mix sensing results for molar ratios i) 1:1:2, ii) 1:2:1, and iii) 2:1:1; the respective polar plot is shown on the right. The red arrow signifies the direction of movement from one VOC balloon to others as the measurement time (converted here to angle = *t* × 10.28) progresses. e,f) Deep learning‐based architecture to predict the chromatogram from typical mix VOCs MVOC1 to MVOC12 using 50 hidden deep learning layers. g) HSGC as the chiral stationary phase. h) Typical sensing result of pure, racemic, and enantiomeric mixtures using mercapto benzoic acid as ligand. i,j) Schematic of the image constructed from layer 2 of the input image patterns generated from combinations of all ligands (FDrGO) for the enantiomeric mixture (2:1 and 1:2), respectively. k) the predicted separation result, and confusion matrix of each ratio is shown with ≈94% accuracy.

**Figure** **3**a–c shows a typical HSGC‐layer‐dependent sensing analysis of a mixture of vapors (Methanol (M): Ethanol (E): Isopropyl alcohol (I) ≈ 1:1:1). As expected, the sensing results in layer 1 shows no separation. However, the response kinetics for the second and third layers shows three distinct peaks, and the effect of the time‐space‐resolved architecture of HSGC is clear. This could be because the porous structure of cellulose modulates the unique mass transfer rate of each molecule in the mixture, so it reaches each layer at a distinct time. Figure 3d shows the complex time‐space‐resolved dataset of mixtures of three volatile organic compounds (VOCs) in various mixing ratios (the radial plot is shown right for each mix condition to show how the relative presence of each VOC component modulate the individual area of each VOC type). For mathematical representation and other information, please see Section S5–S7, Figure S8, Table S3 Supporting Information. Typical predicted mass spectrogram of 24 mixed VOCs using deep learning‐based neural network architecture (Figure 3e,f) shows excellent separation (classification efficiency >95%) and detection (accuracy ≈94 ± 2%) and generation of a continuous chromatogram (see deep learning optimization details Section S8, Figure S9 Supporting Information). The HSGC architecture was tested further for the separation and detection of various mixtures of mirror‐symmetric chiral molecules (enantiomers, e.g., S(+)/R(‐) butanol). These molecules have the same molar mass, so it is highly challenging to split them into racemic/enantiomeric mixtures during the measurement process (see conceptualization in Figure 3g). The results shows that pure S(+) and R (‐) butanol exhibit opposite orientations of resistance‐change modes such as an up–down configuration that suggests strong chiral recognition.

### 2.3 Use Cases of HSGC in Biomedical Applications

#### 2.3.1 Chiral Recognition

Enantio‐separation/detection is important in drug, food additives, clinical research, and medical industries, and is mostly achieved by chromatography and electromigration.^[^ ^12^, ^13^ ^]^ Conventional approaches such as NMR and optical dichroism to rapid detection of chiral compounds in mixtures remain challenging due to the very high cost of separation, long preparation times, data management, and labor intensiveness. Other strategies, such as the use of naturally helical (such as DNA) or synthetically helical (e.g., polymers) sensing structure has been proposed, but they are predominantly limited to strict handling, and sensitive to limited bio‐chemical operating condition.^[^ ^14^, ^15^ ^]^ Considering this challenge, HSGC was tested for its ability to separate/detect mirror‐symmetric chiral molecules (enantiomers, e.g., S(+)/R(‐) butanol) in pure state or as racemic/enantiomeric mixtures. For this, cellulose/lignin fibers^[^ ^16^ ^]^ were used to bind different enantiomers in a spatiotemporal manner, so they can be released and detected later in a distinctive manner (see conceptualization in Figure 3g). As complementary to the spatiotemporal part, hybrid FDrGO sensors were loaded with various chiral/achiral ligands and exhibited a remarkable ability to sense differences between enantiomers.

Figure 3h shows typical response patterns to pure and racemic/enantiomeric mixtures of S(+) butanol and R(‐) butanol from mercaptobenzoic acid ligand as the FDrGO sensor from layer 2 of the HSGC structure. As seen in the figure, excellent chiral recognition and selectivity is achieved between S(+) butanol and R(‐) butanol with opposite direction change. The recognition of their racemic and enantiomeric mixtures through splitting profile suggest a simultaneous chiral separation by the cellulose phase (see Figure 3g). Molecular docking analysis using optimized geometrical configurations of cellulose‐enantiomers (S(+)/R(‐)) (Section S9, Figure S10, Supporting Information) attribute the separation to distinct interaction energies of these two complexes (−146113.24 and −146112.25 kcal mol^−1^, respectively) with the cellulose, possessing a chiral center and naturally helical shape. Using all sensing patterns, a synthetic image (pure/mixture) was constructed and used as input to the self‐learning architecture of the deep neural network (see Figure 3i,j for 2:1 and 1:2 mixtures using all types of FDrGO. As seen in Figure 3k, the synthetic image classifies and quantifies classified and quantified the components very efficiently, integrating temporal‐information, sensor‐chemistry, splitting, and inter‐sensor relationships in a single presentation.

To explain the opposite orientation changes in chiral exposure (S(+)/R(‐)), ab‐initio DFT analyses were conducted for typical case (up‐down, Figure 3h) of 4‐mercaptobenzoic acid‐DrGO as sensor‐host and S(+) and R(‐)) as target guests (see Sections S10–S11, Supporting Information). Optimized structure, HOMO–LUMO gap, and thermodynamic parameters are presented in Figure S11 and Table S4, Supporting Information. Although these calculations revealed the interaction strength of each complex, no further conclusion could be drawn for opposite resistance changes. To bridge this gap, the spin‐polarization effect originating from chirality‐induced spin selectivity (CISS) is considered, where charge injection from the chiral molecule is accompanied by a specific spin orientation ((<↑| or <↓|)) from enantiomers.^[^ ^17^, ^18^, ^19^ ^]^ Owing to specific spin populations, the apparent magnetic structure of the graphitic plane changes its electronic properties markedly (Figure S12b, Supporting Information). This could be attributed to the breaking of local symmetry by N‐doping in C—C lattice during polydopamine encapsulation and reduction of GO to n‐type DrGO (see schematic in Figure S2, Supporting Information) and n‐type FET properties (Figure 2g–l). Due to the specific spin population from adsorbed enantiomers, the N_2_ scattering center on a graphitic plane could act as an effective spin controlling factor and modify the band gap accordingly. The calculated spin‐polarized bandgap, using DFT calculation, shows distinct band modulation. For more discussion of this analysis, see Sections S10–S11, Figure S12–S13, Supporting Information.

#### 2.3.2 Rapid Malignancy Detection and Real‐Time Cancer Spectrogram

Biopsy analysis for cancer detection and classification requires is time‐consuming (scale of hours to days) and requires the skill of a highly trained personnel.^[^ ^20^, ^21^, ^22^, ^23^, ^24^ ^]^ Once the tissue is obtained for biopsy it is preserved in formalin or similar chemicals and transferred to the laboratory. A slide is then fixed and stained with special dyes, such as Hematoxylin and eosin stain (H&E stain or HE stain). The tissue is then frozen into a block of wax or similar substance and examined under the microscope to see the exact structure of the tissue sample and to list any abnormalities or important findings. Other internationally accepted protocols for biopsy analysis include spectroscopic, fluorescence‐based, structural, optoacoustic, and radiological labeling,^[^ ^20^, ^21^, ^22^, ^23^, ^24^ ^]^ which can be projected as an on‐spot coalition among surgeons and pathologists.

**Figure 4:**
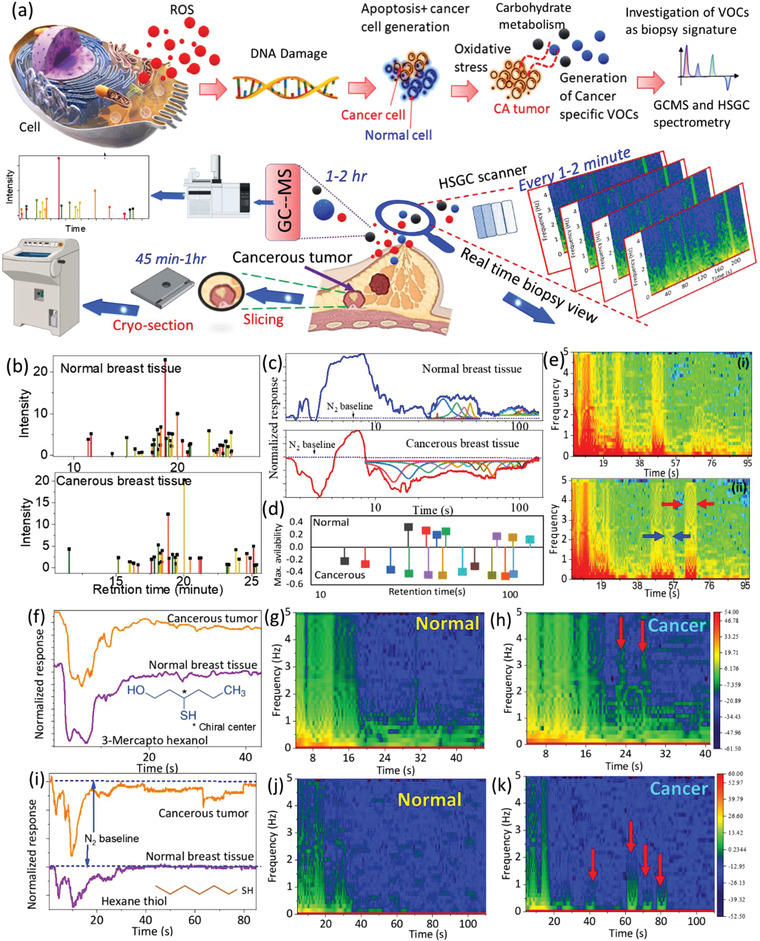
Hybrid Graphene chromatography for rapid cancer diagnosis. a) Schematic model of VOC‐based biomarker (see the mechanism in the inset) analyses for diagnosing breast cancer and comparison of operating time of HSGC device with the cryo‐section procedure during cancer surgery and GC–MS operation. b) The standard GC and c) HSGC‐derived chromatogram of cancerous and healthy breast tissue samples using a typical amine‐DrGO (DrGO‐diethanolamine) sensor. d) The calculated fitted results for estimating retention times to discriminate between cancerous and normal cells with 180° phase difference in retention time (from HSGC sensing result). e) Time‐frequency spectrographic representation of i) healthy breast tissue and ii) malignant tumor. f–k) Effect of sensor ligand chemistry to modulate the spectrum type for healthy and cancerous breast tissues.

Nevertheless, they are often time consuming and complex in nature. HSGC was evaluated against the challenges with the currently available biopsy analysis, so ultra‐fast (1–2 min) analysis could be obtained without the need for staining/biochemical treatments and/or lengthy sample preparation and extensive data analysis. The rationale behind the HSGC‐based approach relies on previous findings that cancerous and healthy tissues are characterized by a distinctive profile of VOCs, due to distinctive lipid peroxidation and carbohydrate metabolism in inflammatory and oxidative stress caused by tumors (see **Figure** **4**a).^[^ ^25^, ^26^, ^27^, ^28^, ^29^ ^]^ Figure 4b shows the standard Gas Chromatography linked with Mass Spectrometry (GC‐MS) result for normal and cancerous tissues. As seen in the figure a clear distinction of the molecular signature is observed for both tissue types. The FDrGO‐derived chromatogram (fitted result) shows distinct retention times and 180° opposite phase‐difference resulting from delayed arrival of electron donating/accepting VOCs at the sensor surface, increasing or decreasing resistance accordingly (Figure 4c‐d). A comparative spectrogram is shown in Figure 4e for faster real‐time detection of malignancy by identifying specific and unique frequency‐band locations (marked arrow) in real time. In spectrogram‐map, a new frequency band either could appear in specific time intervals for cancer tissue (between 51.7 and 57.2 s for 0–5 Hz (blue arrow) and 62.6–98.96 s for 0–3 Hz (red arrow)) or could disappear (between 25 and 28 s) or remains unaltered (44–51 s), when compared with normal tissue. The appearance of the new band could be related to the unique presence of cancer‐specific VOCs that reach the HSGC layer for those specific time frames and remains absent for normal tissues. The excellent distinction between each tissue type is also found for FDrGo‐chemistry dependent spectrum evolution (see Figure 4f–k), causing chemistry‐dependent unique splitted‐time‐resistance profiles with unique spectral features. In each of the cases, the specific band location becomes brighter and appear as a significant strong identificatory of malignant tissue types. For example, hexanethiol‐DrGO shows new band formation ≈40s (0–0.5 Hz), 60s (0–2 Hz), 70s (0–1 Hz), and 80s (0–1.5 Hz) for cancerous tumor tissue (marked red arrow), whereas, for normal tissue, there is no presence of any band formation in this entire time‐period (40–100 s) (see Figure 4j,k). These additional dimensions of sensor‐chemistry dependent spectroscopy could be highly useful in the future for more challenging biological samples, such as determinations of cancer staging, progression/regression of cancer, time evolution of chemotherapy treatment to explore new discovery pathways, and wireless on‐demand drug delivery.^[^ ^30^ ^]^ Additional data for multiple samples and statistical analysis of malignant and non‐malignant breast tissues are shown in Section 12, Figure S14a,b, (Supporting Information).

#### 2.3.3 Wearable Skin Spectrometry

Skin‐derived VOCs carry important information about metabolic processes and are derived from eccrine, sebaceous, and apocrine gland secretions and their interactions with bacterial presence on the skin.^[^ ^31^, ^32^ ^]^ In fact, skin‐emitted VOCs show indications of a variety of diseases, healthcare status, and dietary influence ex vivo.^[^ ^31^, ^32^ ^]^ Although GC–MS‐based approach is extensively used for a variety of clinical studies for identifying specific target VOCs for identification of specific biomarkers, however, not useful for on‐skin real‐time spectroscopic monitoring.

**Figure 5:**
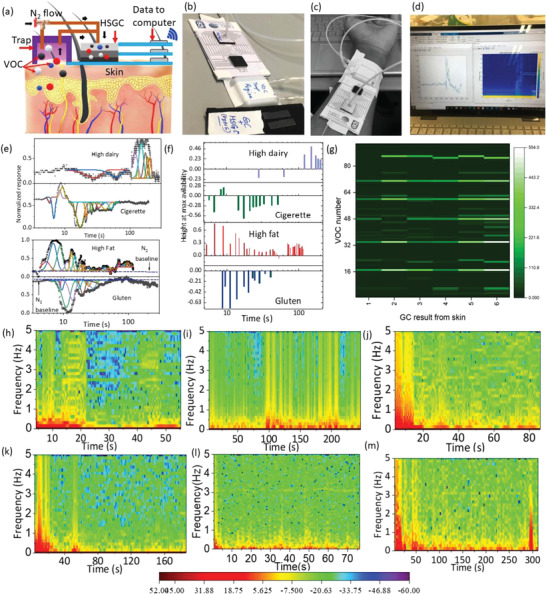
a) Schematic and b,c) photograph of wearable spectrometry from skin implanted HSGC device and trap. d) Real time chromatogram display directly from skin measurements, e) Chromatogram of skin extract obtained from HSGC geometry to monitor dietary influences, and f) related retention times and heights showing maximum abundance. g) GC‐MS heat map results, and h–m) HSGC extracted chromatogram to monitor dietary influences (gluten (1), caffeine (2), dairy (3), fatty meal (4), cigarette smoke(5), and sugary product(6) (see details also in Table S5 and Figure S15, Supporting Information).

Stacks of HSGC were used as wearable spectrometer for analysis of various dietry influences via skin (see **Figure** **5**a–d). In‐situ measurements show distinctive separation and chromatogram of VOCs, whose composition in the headspace of skin was changed after various dietary intakes (see Figure 5e,f). The standard GC heatmap and HSGC‐spectrogram for each type of dietary influence (such as gluten, smoking, sugar) are shown in Figure 5g–m. The GC‐MS benchmark results and list of VOCs are shown in Section 13, Figure S15 and Table S5, Supporting Information. The relative changes in the heatmap brightness show that the spectrum evolved directly from the skin for each dietary type is significantly different from each other (both from extracted time stamp (Figure 5f) as well as a spectrogram (Figure 5h–m)). These changes are consistent with those observed by lab‐based GC‐MS. The significant difference for each case could be attributed to biological (due to variation of metabolic activity) and behavioral sources (dietary intake). In the spectrogram map, for example, the brighter band appears in the range of 100–200 s (0–5 Hz) for caffeine and ≈300 s (0–3 Hz) for sugary intake. This spectral feature could be used to see how this color band moves, shift and appear/disappear for a variety of target applications. Essentially, the spectral map shows not only unique band formation in a specific “time sequence” but also modulates the “frequency bandwidth” that represents the variation of frequency harmonic information from the influence of various molecules secreted from the skin. This is also observed for cancer specimen analysis. The wearable devices for monitoring the skin for detection and prognosis of diseases and health status spectrophotometrically ex vivo are therefore feasible. A demonstration video showing on‐skin spectrogram measurements using an implanted HSGC on the skin (human arm) is provided in Video S2, Supporting Information. In a future of “do it yourself” (DIY),^[^ ^33^ ^]^ this high precision, low cost, and flexible wearable chromatography‐based micro device would be highly desirable for body‐area network protocol where multiple devices could be worn together in various parts of the body (skin) through the interconnected wireless mode. This could have broad applications for fitness tracking, 24 h healthcare monitoring, supervising forbidden drug consumption, and connected personal healthcare.

## 3 Summary and Conclusions

HSGC was designed, fabricated, characterized and implemented in various biomedical applications. The HSGC relies on a time‐space resolved geometry with a cellulose‐based membrane that induces time lags of various compounds found in a complex mixture, similar to conventional gas chromatography; and an array of sensors that detect each eluted compound by fingerprint of electrical/digital signals empowered with deep neural network models. It could be used as a unique platform for recognizing a wide variety of compounds in complex mixtures and would be highly suitable for molecular spectroscopy‐based diagnostics in various medical applications. Using the specific frequency‐band formation in the spectral map could provide unique time sequences as well as frequency‐band modulation by adding or subtracting specific harmonics information from the interaction kinetics of ultra‐thin FDrGO chemistry and a variety of unique molecular presence in the targeted samples. Implementing deep‐neural‐network and synthetic‐image‐processing, exhibited a highly promising results for medical diagnosis and drug industries (enantiomer/racemic mixture evaluation using spin‐sensitivity (CISS)). Robust chiral discrimination, spin‐sensitivity, and magnetic influence could open new frontiers in chiral drug research, chiral‐spintronics/spin logic, and magnet‐free memory applications. The reported HSGC approach could be used as a micro‐chromatography electronic pen for surgeons for rapid‐biopsy (1–2 min) of malignant lesions during on‐site surgery as an alternative to cryosection and as a wearable spectrometer for health‐care monitoring for future DIY body‐area‐network protocol. The strategy could be useful for many other exciting bio‐medical device research and therapeutic‐electronics applications, such as time‐evolution monitoring of chemotherapy treatment (in vitro/in vivo), rapid cancer staging, cancer prognosis and developing chromatographic‐breath‐analyzer.

## 4 Experimental Section

### Synthesis and Binder‐Free Inkjet Printing Recipe for Hybrid FDrGO Ink

In a typical synthesis, 100 mg of GO and 50 mg of dopamine hydrochloride (Sigma) were added to 200 mL of 10 mm Tris solution (pH ≈ 8.6) and sonicated for 15 min in an ice‐cold water bath for dispersion. The reaction mixture was stirred at 400 rpm at 65 °C for 24 h for reduction (rGO) and subsequent encapsulation by polymerization (DrGO) through the Michel addition or Schiff mechanism (see detailed reaction schemes Figure S2, Supporting Information). The product (DrGO) was cooled to room temperature and washed ≈10 times with DI water and ≈5 times with ethanol and collected by centrifugation. In each water wash, the pH of the medium was checked until it reached pH ≈ 7. The cleaned and dried DrGO powder (5 mg) was dispersed in 20 mL DI water pH ≈8.6 (adjusted with Trizma) and mixed with 10 mg amino‐terminated or 20 mg thiol‐terminated chiral/achiral ligand (see the list in Table S1, Supporting Information). For water‐insoluble ligands, 1:1 water: ethanol was used as the dispersion medium. The reaction mixtures were gently stirred for ≈15 h at room temperature, then washed 10 times with water and 5 times with ethanol, collected centrifugally, and dried under reduced pressure. The pH was checked at each washing step as before until neutrality was attained. The dried functionalized powder (FDrGO) was redispersed in DMF and sonicated at low power for ≈1–2 min to disperse the ink, subsequently used for inkjet printing (Dispensing system with piezoelectric nozzle (Scienion, model sciFLEXARRAYER S3)) on paper. The details of the binder‐free print approach, printing on a wide range of substrates, reaction mechanism, list of biochemical ligands (thiols, amines, chiral compounds), and its advantage over conventional printing from the literature are discussed Section S1 and S2, Supporting Information.

### Silver Nanowire Synthesis

1D silver nanowire was made using the solvothermal seed‐mediated method. In view of its photosensitivity, the AgCl seed solution was prepared in the dark. Typically, aqueous silver nitrate (5 mL, 0.5 m) was mixed with sodium chloride solution (5 mL, 1 m) and stirred at ≈700 rpm for 1 min. Within a few moments, white silver chloride precipitated and was separated, washed with deionized water, and kept under a vacuum for 1 h in the dark. Next, 1.36 g of PVP (MW 40 000) was dissolved in 160 mL ethylene glycol in a round flask (with reflux) at 700 rpm at ≈170 °C for 5 min. An excess (≈50 mg) of the dried fine AgCl powder was added to this hot solution and within 1–2 min the solution became light yellow, indicating the formation of a very fine silver NP. After 3–4 min, 0.22 g AgNO_3_ was added and the mixture was stirred at 300 r.p.m. The solution color changed in the sequence of light yellow, dark yellow, brown, green, and finally gray (see Figure S4, Supporting Information). This indicates the formation of long silver nanowires. The solution was cooled to room temperature and the product was cleaned with water and ethanol washing 10 times and centrifugation to remove extra PVP and EG. The fresh cleaned silver nanowire was then dispersed in isopropanol with mild ultra‐sonification for 30 s and used for inkjet printing on paper.

### Characterization of GO, DrGO, FDrGO Inks (SEM, Raman, FTIR, and NMR Analyses)

A morphological study using SEM (Sigma 500, Zeiss Ultra‐Plus High‐Resolution SEM, Germany) revealed a microscopic view of the synthesized materials dispersed in alcohol and spin‐coated on silicon wafers (Si/SiO_2_) to distribute the flakes uniformly. Images were acquired at ≈5 kV electron acceleration and 4–8 mm working distances. Raman spectra (Horiba Jobin Yvon LabRAM HR Evolution Micro‐Raman, Japan) were obtained with a 532 nm laser. All samples dispersed in ethanol were spin‐coated on silicon wafers and dried overnight in a vacuum chamber before measurements. For FTIR (Bruker Vertex 70 V KBr BS vertical ATR‐FTIR, USA) measurements (300–4000 cm^−1^), the cleaned and overnight‐dried sample powder was mixed with KBr to form a pellet. 1H NMR analysis was done with a Bruker AV‐III400 MHz two‐channel spectrometer with a direct detection probe based on automatic tuning and matching (equipped with *z* gradients) at room temperature using ≈500 µL d‐DMSO (Sigma–Aldrich, USA) as the solvent at 30°; 1 H pulse with 1 s repetition.

### Electrical Characterization

For typical gate‐dependent FET properties for determining carrier type, DrGO/FDrGO samples were spin‐coated on Si/SiO2 wafers with an electrometer (Keithley 2636A and 3706, USA) interfaced with Labview software, USA.

### Cell Culture Treatment with FDrGO Inks to Assess Cytotoxicity

Human epithelial lung cells (BEAS‐2B, ATCC, CRL‐9609, Israel) were seeded at 96 × 10^3^ cells per well on 24‐well plates and cultured with 0.5 mL full RPMI‐1640 medium (Sigma‐Aldrich, Israel) at 37 °C with 5% CO_2_. After 24 h recovery, the cells were washed with PBS Ca^2+/^Mg^2+^ (Phosphate Buffered Saline, Sigma–Aldrich, Israel) and the treatments were applied for 24 h in the cell culture full medium. FDrGO samples were vortexed shortly before making final dilutions for treatments. Cells were exposed to 10 and 100 µg mL^−1^ FDrGO.

### Annexin V–FITC/PI Assay

After 24 h treatment at the indicated concentrations, cells were gently washed with PBS. Annexin‐V and propidium iodide (PI) staining were performed according to the manufacturer's instructions (BioLegend, California, US) in 0.2 mL full RPMI‐1640 medium per well. The cells were again gently washed with PBS, and 0.2 µg mL^−1^ Hoechst 33 342 solutions (Invitrogen by Thermo‐Fisher Scientific, Israel) was added to them. The cells were observed and analyzed using an In Cell Analyzer 2000 System (Technion Life Sciences and Engineering Infrastructure Center, Technion, Israel).

### Preparation of Breast Cancer and Normal Breast Tissue Samples

Frozen cancerous and healthy breast tissues from different patients were purchased from the Israeli Biorepository Network for Research MIDGAM (Haifa, Israel). Both cancerous and noncancerous tissues from patients who had not undergone chemotherapy were collected after surgery. The frozen tissues were transported inside plastic cryovials in dried ice and kept at −80 °C pending the experiment. All tissue samples were thawed in their plastic cryovials to room temperature for 10–20 min and ≈40 mg samples were immersed (500 [µL]‐ tissue [mg]) µL in 0.9% NaCl in double‐distilled water. EPA 524 internal standard mix with 2000 µg mL^−1^ 1,4‐dichlorobenzene‐d4 in methanol, purchased from Sigma‐Aldrich, was used as internal standard at 40 ppb. Tissue samples were analyzed by GC‐MS and the HSGC sensor set‐up.

### GC and HSGC Sensor Measurements of Malignant/Non‐Malignant Tissues

Chromatographic analysis involved a GC‐7890B/MS‐5977A instrument (Agilent) equipped with an SLB‐5 ms capillary column (30 m length; 0.25 mm internal diameter; 1 µm thickness; Sigma Aldrich) combined with an ITEX PAL RSI 120 headspace autosampler system. Trap preclean conditions were 180 s and 260 °C. Incubation of samples was set at 55 °C for 15 min. Agitator speed was defined as 500 rpm. Desorption flow was 10 µL s^−1^ at 250 °C, while extraction strokes were set at 100, extraction volume at 1000 µL, and aspirate and dispense flow rates at 100 µL s^−1^. The injection aspirate flow rate was defined as 10 µL s^−1^. The carrier gas was helium, the flow through the column was set at 1.5 mL min^−1^ and the temperature in the column ranged between 35 °C and 300 °C at a rate of 15 °C min^−1^. The GC‐MS chromatograms were analyzed using Mass Hunter qualitative analysis version B.07.00, Agilent Technologies, USA, and spectral library match NIST was used to identify compounds. HSGC measurements of all clinical samples were conducted using a 55 °C hot water bath and ultra‐pure N2 as a baseline with ≈10 Hz sampling frequency using an Arduino Mega board and I2C interconnected protocol.

### Spectrography of Cancerous/Healthy Tissue and Wearable Skin‐Based Analysis

Typical HSGC results in the time domain (see Figure 4c) obtained from clinical samples were transformed into the frequency domain (Figure 4e) with a short time window‐based FFT algorithm using Hanning. The biggest spectrogram differences between samples show time positions at which specific VOCs appear that are distinctive of malignant/nonmalignant samples. This robust technique (1–2 s) could be used during continuous data sampling and enabled identification to be made in real time through color‐mapping or color‐band representation (see Figure 4e). A demonstration of on‐skin spectrum monitoring using the HSGC approach is shown in Video S2, Supporting Information. Note that for GC‐based skin VOC extraction, we used a PDMS‐based VOC absorber attached to the skin in parallel during on‐skin spectral monitoring. Later this skin‐attached PDMS absorber was carefully taken off using suitable tweezers and kept inside the N_2_ cleaned vial for GC measurements. In this way, standard measurements from GC and direct skin measurements could be correlated for future use and deterministic calibration.

## Conflict of Interest

The authors declare no conflict of interest.

## Supporting information

Supporting Information

Supplemental Video 1

Supplemental Video 2

## Data Availability

The data that support the findings of this study are available in the supplementary material of this article.
