# Supplementary material for: Ultra‐Fast Portable and Wearable Sensing Design for Continuous and Wide‐Spectrum Molecular Analysis and Diagnostics
Source: Adv Sci (Weinh). 2022 Oct 20;9(34):2203693. doi: 10.1002/advs.202203693 (PMC9731699; doi:10.1002/advs.202203693)
Supplement: Supplementary file 1 — Supporting Information [file ADVS-9-2203693-s002.pdf]

Supplementary Information:

Ultra-Fast Portable and Wearable Spatiotemporal Sensing Device for Continuous and Wide-Spectrum Molecular Analysis and Diagnostics

Arnab Maity,[1] Yana Milyutin,[1] Vivian Darsa Maidantchik,[1] Yael Hershkovitz Pollak,[1] Yoav Broza,[1] Rawan Omar,[1] Youbin Zheng,[1] Walaa Saliba,[1] Tan-Phat Huynh,[2] and Hossam Haick[1*]

[1] *Department of Chemical Engineering and Russell Berrie Nanotechnology Institute, Technion - Israel Institute of Technology, 3200003, Haifa, Israel*
[2] *Laboratory of Molecular Science and Engineering, Faculty of Science and Engineering, Åbo Akademi University, Henrikinkatu 2, FI-20500, Turku, Finland*

[*] *Corresponding author (H.H.): hhossam@technion.ac.il*

This PDF file includes:
Supplementary Figures S1- S15
Tables S1 - S5
Video S1: Inkjet printing video of functionalized graphene ink on paper.
Video S2: On-skin direct measurement of molecular spectroscopy using HSGC structure.

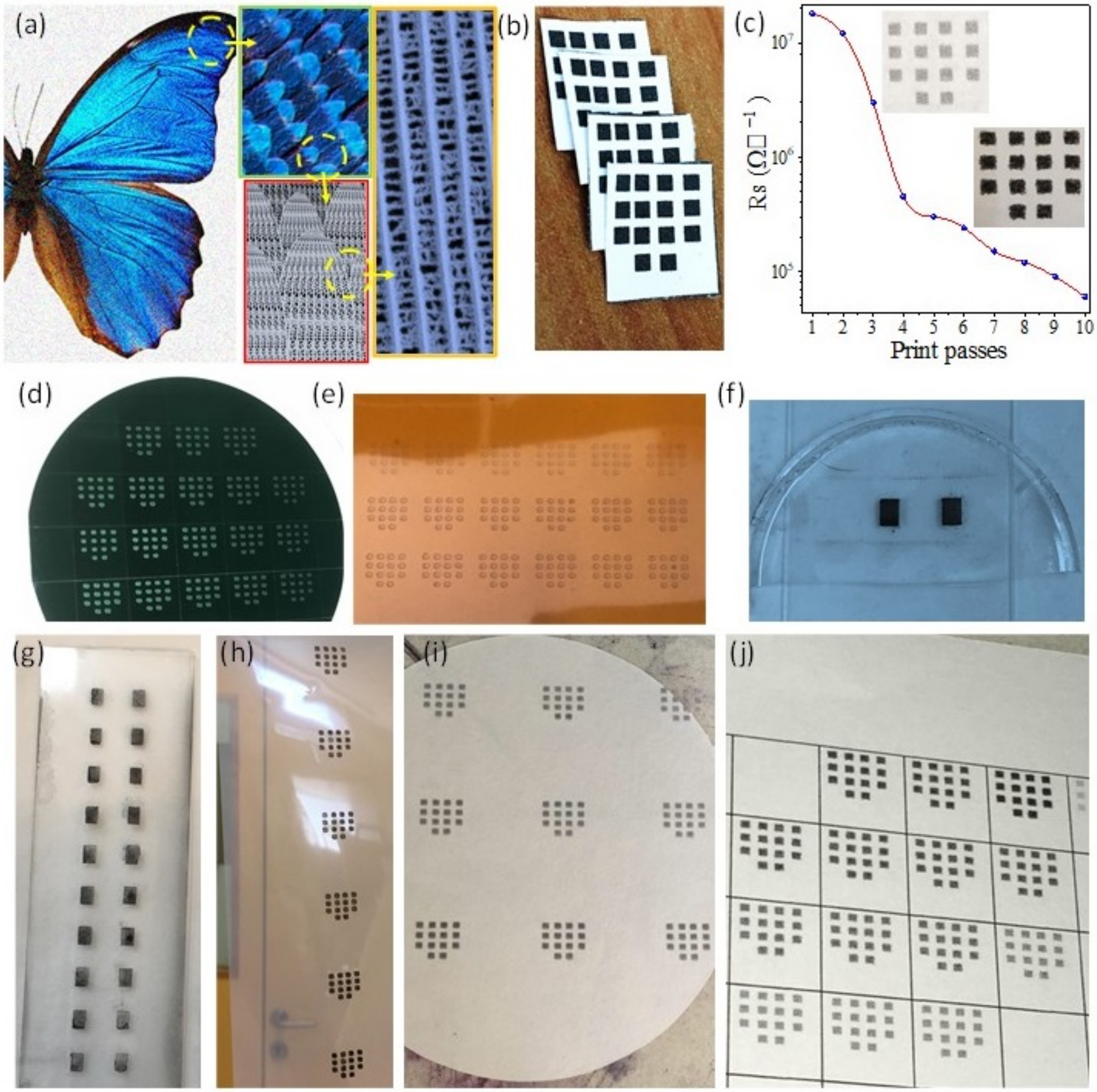

Figure S1. Bio-inspired design of HSGC and large-scale fabrication of sensor. The micro-structural similarity between **a)** butterfly wing arrangements (layer by layer configuration and its porous structure) and **b)** hierarchical printed paper. **c)** Typical resistance changes for various print pass of DrGO ink. Photograph of mass scale printing of the hybrid graphene ink (DrGO) on various untreated substrates, such as, **d)** Si/SiO_2 wafer, **e)** Kapton, **f)** PDMS rubber polymer, **g)** Glass, **h)** Transparent PET**, i)** Wattman Filter paper and j) Normal office paper.

S1. Synthesis and Binder-Free Inkjet Printing Recipe of Hybrid Graphene Ink

Dopamine is biochemically signified as hormone and neurotransmitter inside the human body, and it transforms to polydopamine through self-polymerization in weak alkaline media which served as simultaneous reducing agent and surfactant during the reduction of GO to rGO. The catechol groups of Polydopamine coated rGO (DrGO) could be oxidized to quinone form in weak alkaline condition and further could be used to functionalize with thiol or amine containing compounds either by Michel addition or Schiff based mechanism.[1] Then the powder (DrGO) is dried in vacuum for functionalization (*see* Methods Section) This versatile reaction pathway has been used to nano-engineer the grafting process for tuning the surface chemistry of graphene plane with variety of multiple chiral and achiral ligands for constructing inkjet-printed grade sensor array which is connected through printed silver nanowires prepared with a rapid solvothermal chemical route. Note that present rGO or rGO-ligand ink formulations do not require any additional stabilizing agent or binder (thus no post printing annealing is required for surfactant/binder removal) and could be readily dispersed in various organic solvents to print the different geometry on rigid, flexible and porous substrates (*e.g.,* Si/SiO_2, Kapton, glass, PET, silicone rubber, papers (*see* Figure S1) by simply modulating the printer parameters for film deposition in layer by layer approach The printed pixel geometry shows excellent boundary uniformity without any visible coffee ring effect.

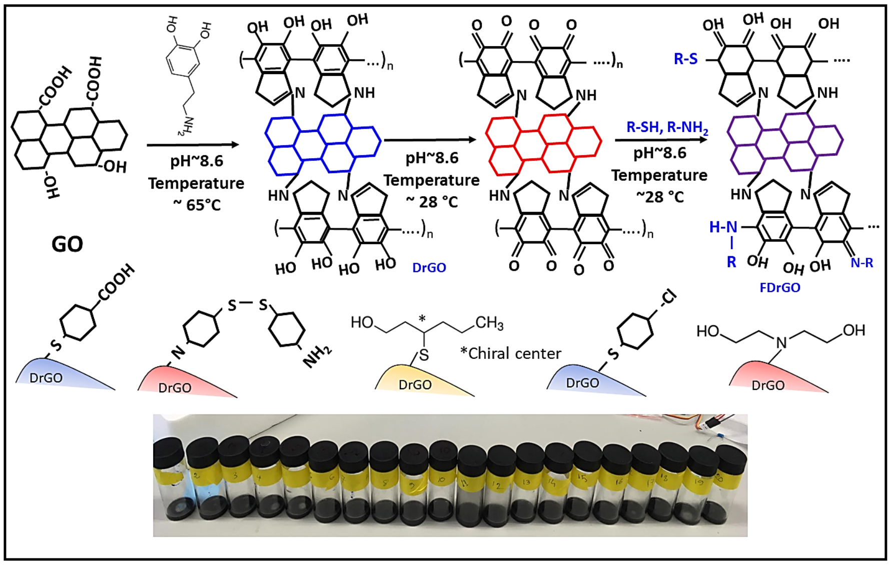

Figure S2. Reaction scheme. Synthesis strategy of simultaneous reduction, encapsulation, and dispersion of DrGO based hybrid ink and its functionalization (FDrGO) with various amine/thiol-based ligands. Optical image of 20 different FDrGO inks dispersed in DMF (see the list below).

S1.1 Hybrid ink of dopamine-rGO and related functionalization with chiral/achiral ligand

To get a stable suspension of graphene ink for uniform printing geometry without nozzle blockages, various attempts have been reported.[2] These include use of stabilizer/surfactant (such as, Triton, pyrene sulfonic acid) for very good dispersion of the ink, adjustment of desired viscosity of the ink without any coffee ring effect by suitably chosen dispersion media (such as, NMP, terpineol, alcohol), surface tension matching between substrate and ink droplet by tuning the hydrophobicity (such as, spin coating of

bis(trimethylsilyl)amine (HMDS) or hydrophilicity (plasma) of the surface, use of binders and additives (such as, PVP, ethyl cellulose, PEDOT:PSS) for improvement of the post printing adhesion between the ink and the substrate.[3] Although the use of stabilizer promotes better graphene stabilization, but its tedious to remove the excess stabilizer from the ink for getting the better conductivity. Similarly, use of binder/additives may promote better adhesion for wide range substrate, but again post-printing thermal annealing is needed to remove extra adhesives, and this high temperature treatment is not suitable for various flexible substrate like paper/plastic. In this scenario, dopamine could be a better alternative as this specific material could be used as direct solvent mediated reducing agent to remove oxygen containing functional group from graphene oxide (GO) to convert into reduced graphene oxide (rGO), as well as it serves as surfactant through the formation of polydopamine by in-situ polymerization on rGO surface (see Figure S2). This hybrid system (rGO-polydopamine (DrGO)) is stable in water and various organic solvents (such as, dimethyl formamide (DMF), chloroform) and readily available for inkjet printing without using any additional surfactant, thereby, no need for post-printing high temperature annealing. In addition, due to the presence of catechol group inside the DrGO nano hybrid, the adhesion property is very similar to natural adhesive mussel protein (enriched with amines and catechol group) that has higher affinity to bind with wide range organic/inorganic substrates, such as metal/metal oxides, polymers[3]. This additional inherent adhesion property of the DrGO hybrid could be an excellent alternative for an additive/binder-free printing technology without using any tedious viscosity adjustments, such as, using high boiling point solvent, thus, suitable for printing with wide range substrate application.

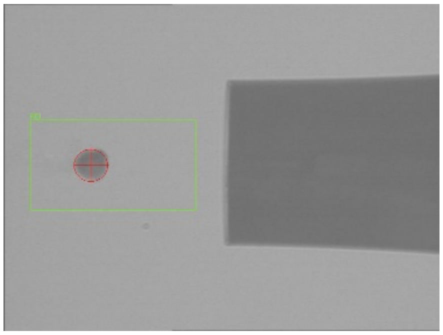

Figure S3. Realtime snapshot of ink. The photograph of DrGO droplet (dispersed in DMF) jetting during the printing. No satellite droplet was found.

Figure S3 shows the real time capture footage of DrGO droplet dispersed in DMF without any satellite droplets and therefore printed geometry provides excellent boundary uniformity as discussed before. As prepared cleaned and dried DrGO powders are then used for grafting reaction to bridge various thiols, amines, and chiral compounds to make FDrGO powders (see method for details of the synthesis process) The dried functionalized powder (FDrGO) is redispersed in DMF and sonicated at low power for dispersion of the ink for ~1-2 minutes and subsequently used for inkjet printing on paper (Figure 1a-b in the article). The list of biochemical ligands used in this study are provided in Table S1. A comparative printing approach for present binder-free printing and state-of-art is provided in Table S2.

S1.2 Biochemical ligands for synthesis of functionalized hybrid ink

Table S1 | List of thiol and amine terminated various achiral and chiral ligand used for constructing FDrGO based multifunctional ink

Amine based ligands	Thiol based ligands
Aniline	Mercapto propionic acid
Diethanolamine	3-Mercapto hexanol
Ethylene diamine	Mercapto ethanol
4-4' di amino diphenyl methane	Mer capto benzoic acid
Butylamine	4 amine phenyldisuplhide
Polyethyleneimine	2-amino 4-chloro benzene thiol
1,4 Phenyladimine	4-chloro benzene methane thiol
Diethylamine	2-napthalenethiol
Oleylamine	4-tert butylebnezene thiol
Cysteine	Hexane thiol

S1.3 Comparison between the reported printing approach and state-of-art

Table S2 | Benchmarking of the chemicals and components used for traditional inkjet-printed graphene-based materials and our work

Binders additives	Substrate treatment before print	Post printing annealing	Substrates	Solvent	Device used	Reference
Ethyl cellulose	Yes	Yes	PET	NMP, Terpineol/CHO	Photodetectors	4
PVP	Yes	Yes	Si3N4	IPA	Gas sensors	5
PEDOT:PSS	No	No	Carbon	Water	Gas sensors	6
Plasdone S-630	No	Yes	Paper	IPA/n-butanol	Conductive inks	7
Ethyl lactate/octyl acetate/ethylene	Yes	Yes	Nitrocellulose	Glass, Kapton	Conductive inks	8
Poly-dopamine (surfactant+ binder) dual	No	No	Paper, Silicon wafer, Glass, Kapton, PET, PDMS	DMF, IPA	Hybrid ink printed sensor	**This work**

S2. Synthesis of Silver Nanowire for Constructing Flexible Electrodes on Paper

One-dimensional silver nanowires are synthesized using solvothermal seed mediate method.[9] The details of the synthesis process, operating temperature and cleaning procedure has been provided in Methods Section of the article. The typical color change during the solvothermal reaction is shown in Figure S4.

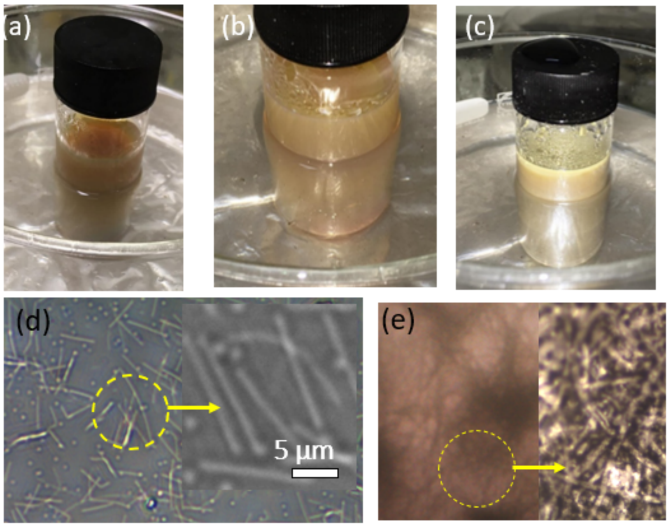

Figure S4. a-c) Color change during Ag NW synthesis. The color change during silver nano wire growth (as time progresses from left to right. The typical microstructure is shown for **d)** deposited nano wires and **e)** sprayed on paper.

S3. Bending Test of Flexible Silver Nanowire-Based Electrodes

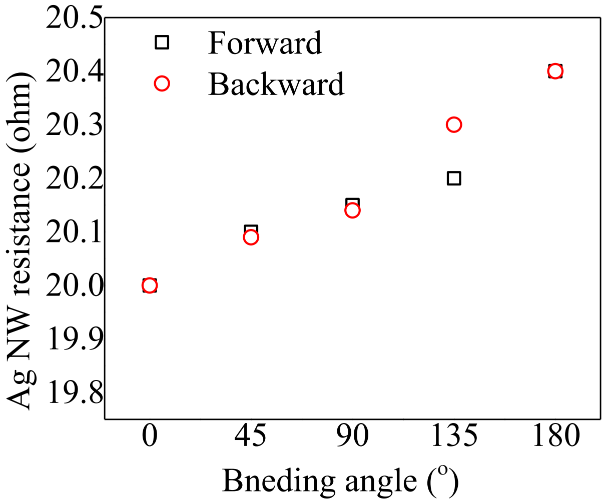

Figure S5. Resistance changes upon bending. The resistance changes of silver nanowire printed conducting line for various bending angles.

S4. Characterizations

S4.1 SEM analysis

Figure2 a-d shows the typical SEM micro-structure of GO, DrGO, thiol-FDrGO and amine-FDrGO respectively with an impression that nano-structural flakes maintain almost 2D flat structure after reduction (DrGO) and functionalization (FDrGO) with various thiols/amines/chiral/achiral ligands.

S4.2 Raman analysis

Figure 2e shows Raman spectrum plot of GO, DrGO and typical FDrGO samples (**thiols**~ DrGO-2 amino-4cloro-benzen thiol, **amines** ~ DrGO- diethanolamine, **chiral**~DrGO-3-Mercapto-hexanol) with calculated I_D/I_G values. Here, G band~1571 cm^{-1} and D band ~ 1346 cm^{-1} correspond to E_{2g} phonon of sp^2 C atoms and breathing mode vibrations of κ points phonons (A_{1g} symmetry)) respectively. An increase of I_D/I_G values suggest the effective reduction of GO (0.28) to DrGO (~0.8). The calculated values of FDrgO samples are spanned between 0.3-0.7.

S4.3 FTIR analysis

Figure 2f shows the typical FTIR spectrums of GO, DrGO and typical FDrGO samples (**thiols**~ DrGO-2 amino-4cloro-benzen thiol, **amines** ~ DrGO- diethanolamine, **chiral**~DrGO-3-Mercapto-hexanol). The emergence of intense absorption peaks at ~1096-1100 cm^{-1} (C-O-C stretching) after functionalization (FDrGOs) suggests successful integration of ligands during polymerization.

S4.4 NMR analysis

Figure S6 shows comparative ^{1}H NMR spectrum for FDrGO samples (**thiols**~ DrGO-2 amino-4cloro-benzen thiol, **amines** ~ DrGO- diethanolamine, **chiral**~DrGO-3-Mercapto-hexanol). It is found that distinct peak shift is observed for typical thiol (3.338 ppm) and amine (3.344 ppm) based FDrGO from DrGO sample (~3.36 ppm).

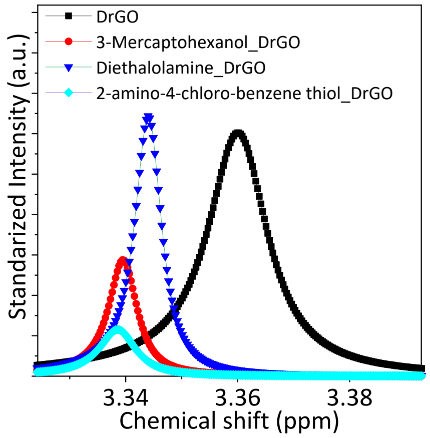

Figure S6. ^{1}HNMR spectroscopy for estimating surface chemistry loading for various samples.

S4.5 Electronic characterization

Typical FET transfer characteristics and I-V measurements are shown in Figure 2g-l for DrGO and typical FDrgOs. I-V and FET characteristics respectively shows ohmic nature of the contact and n-type semi-conductivity for which could be due to integration N_2 adatom at graphitic plane from polydopamine polymerization (reduction + encapsulation) on graphitic lattice. The influence of N_2 adatom in its electronic band gap is later discussed later in DFT calculation (see Sections S10-S12 below). The schematic of the FET measurement platform is shown in Figure S7a below.

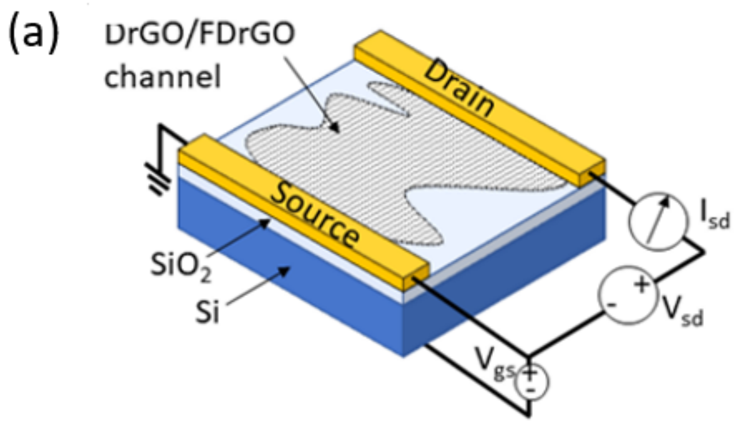

Figure S7. Schematic of FET measurement platform for electronic characterization.

S4.6 Biocompatibility analysis

Cytotoxic assessments are performed for human epithelial lung cells (*see* Figure 1g-r in article) using various FDrGO inks (**thiols**~ DrGO-2 amino-4cloro-benzen thiol, **amines** ~ DrGO- diethanolamine) for 10-100 µg/ml dosage for 24 hrs treatment time (see Figure 1m in article). In Cell Analyzer 2000 System was used to assess the viability of human bronchial epithelial cells (BEAS-2B) using cellular markers of apoptosis (Annexin V-FITC), necrosis (propidium iodide - PI), and nucleus (Hoechst). Annexin V attaches to phosphatidylserine (PS) present on the cell membrane during early apoptosis, whereas PI indicates loss of membrane integrity during late apoptosis or necrosis. The low percentages in the samples of early apoptosis, late apoptosis, and necrosis resulted from the cell washing protocol for microscopy. For this reason, we varied the number of cells and the number of live cells to characterize the cytotoxicity of FDrGOs at different concentrations. Figure 1r in article shows plot of calculated live cell% (Data are represented as mean ± SE (untreated n=3, treated n = 3, error bars indicating variations inside each set of triplicates respectively). Based on these results, it is found that at low concentration cell death is marginal but at high concentration there is a decrease in the number of live cells. Viability was calculated by dividing the treated number of live cells against the untreated number of live cells. Measured calculation shows the ratio in the range of 86.7-88.3% at low concentration and 67.3-74.1% at high concentration. It suggests a guideline for working with these materials in continuous synthesis, production, printing line in industrial arrangement as per maximum permissible intake. Details of the cell culture procedure, FDrGO treatments and assay preparation for imaging are provided in the method section in the article.

S5. Comparative Sensing study of Direct and HSGC Exposure

To evaluate the performance of HSGC, a single-layer array including functionalized sensors was examined upon direct exposure (DE) to various chemical structural/chiral mixtures, without a spatiotemporal part (Figure 1d (inset) in the article). Representative sensing results, such as those for methanol (6.8-13.9 ppb) and pure/mixed vapors of ethanol and isopropanol (M:E:I~0:0:1, 0:1:0, 1:0:0, 1:1:1, 2:1:1, 1:2:1, 1:1:2), are shown in Figure S8a-c. As seen in that figure, DE provided superposed exponential response/recovery kinetics for overall mixture, but no further information could be obtained about each alcohol component. This superposed response could originate from infinite range of possible ratios of components in mixture, which would generate the same superposed exponential response kinetics every time, each component contributing. This issue becomes more challenging when number of molecules in mixture increases. In addition, it would be very difficult to train an intelligent system to select from the infinite range of possible ratios in real sample analyses. The HSGC approach changes the situation dramatically.

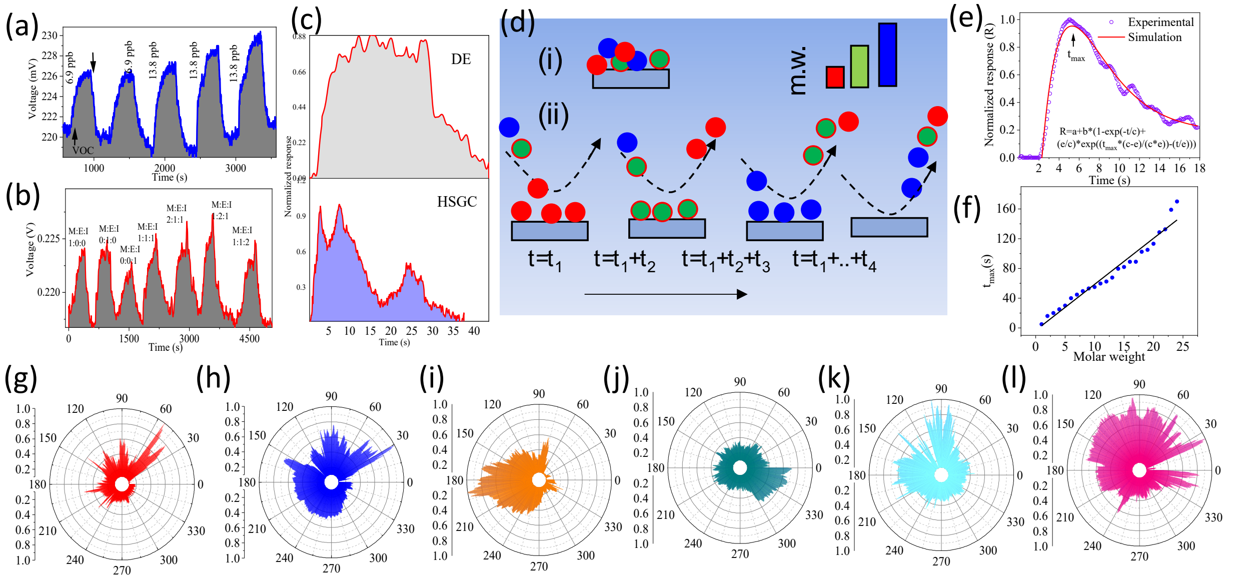

Figure S8. Comparison between DE and HSGC exposure**.** Typical direct exposure sensing performance of a) methanol (6.9-13.8 ppb), b) other alcohol mixing (Methanol (M), Ethanol (E) and Isopropanol (I) for 6.9 ppb in pure (e.g. 6.9: 0:0) and multiplied in the mixture in the ratio of 1:0:0, 0:1:0, 0:0:1, 2:1:1, 1:2:1, 1:1:2. c) Comparative sensing performance of typical 1:1:1 mix vapors (E:M:I) for DE and HSGC exposure at layer 2. d), Schematic showing effects of (i) direct exposure and (ii) time-space-resolved exposure from the unique HSGC structure, where each component (distinct molecular weight) reaches and leaves the sensor surface at a different time (t1, t1+t2,.. , t1+t2+t3+t4). e) Experimental (dotted) and simulated (continuous line) results for measuring tmax. f) Calibration of t_{max} and molecular weight at layer 2. g-l) Layer 2 HSGC sensing (polar representation) for mixtures of 24 vapors: alcohol rich (*e.g.*, ethanol: other VOC ~ 10:1); ketone rich (*e.g.*, acetone: other VOC ~10:1); aldehyde rich (heptaldehyde: other ~ 10:1); organic acid rich (heptanoic acid: other VOC ~ 10:1); hydrocarbon rich (decane: other VOC ~ 10:1); and benzene derivative rich (dichlorobenzene: other VOC ~ 10:1), respectively.

Figure 3a-c in the article shows an HSGC-layer-dependent sensing analysis of a typical mixture of vapors (M:E:I~1:1:1) for layers 1-3 (position of measurement layer is highlighted deep blue in the inset). As expected, results showed no separation in layer 1. However, for 2nd and 3rd layers, response kinetics shows

three distinct peaks with a clear impression of time-space-resolved-architecture of HSGC. This simultaneous separation and sensing capability arise from porous structure of cellulose network within HSGC architecture, which modulates unique mass transfer rate of each molecule in mixture so that they reach each layer separately, thereby creating three major peaks and areas – *cf.* fitted colors: methanol (red); ethanol (purple); isopropanol (blue). To identify each peak, the sensing of other vapor combination ratios was examined (1:1:2, 2:1:1, 1:1:2). In manuscript Figure 2d(i)-(ii) present results from HSGC layer 2 as a representative example: the area or peak specific to a particular analyte (volatile organic compound; VOC) is sensitive to composition ratio and increases or decreases according to nature of the applied mixture (see also radial balloon plot on the right (Figure 2d in the article) for relative contributions of components in the mixture as the angle increases from 0 to 360° (time converted to angle (r) = t*10.28, where t = time in seconds)). Therefore, HSGC-based LE approach is clearly more informative, because different components enter, interact with, and leave each specific layer at different times (see schematic in Figure S10d(ii)), whereas the DE exposure approach (schematic in Figure S10d (i)) shows collective effect of all components over measurement time. The two sets of response kinetics are compared in Figure S8c. The response-recovery kinetics is modeled using Langmuir adsorption kinetics, as shown in HSGC scheme, Figure S8d (ii). The equation was constructed to describe the processes of entering, interacting with, and leaving a particular layer. Figure S8e shows the measured (dotted) and fitted (continuous) curves obtained using the equation:

$$R(t) = a + b.\left(1 - e^{-\frac{t}{c}}\right) + \frac{e}{c}.\, e^{t_{max}\frac{c-e}{c.e} - t/e} \qquad \text{(S1)}$$

where R(t) is continuous measurement of sensor resistance at a certain time (t), t_{max} is the time at which resistance is maximum for a specific analyte (different for different analytes), and a, b, c, e are kinetic constants, adsorption, and desorption parameters (*see* details in Section S6). Owing to differences in adsorption and desorption energies (c and e in Equation S1), fitted sensing profile is asymmetric and broad (see Figure S8e). For multiple analytes in a specific mixture, Equation S1 could be superposed with distinct t_{max}, b, c, e values (see Section S6 below). Figure S8f shows typical calculated t_{max} values for different analytes at layer 2, demonstrating that t_{max} varies with molar masses of VOCs. HSGC was further challenged with more complex mixtures of 24 analyte types (alcohols, aldehydes, ketones, hydrocarbons, organic acids; see Section S8 and Figure S8g-l for a polar plot of typical results). Whole complex data set was used as sequential input for deep learning (s*ee* Figure 3e in the article) to generate continuous chromatograms of typical mixture samples MVOC1-12 (*see* Figure 3f in the article). Details of input generation, hidden layer optimization and data compression using wavelet-based signal processing are provided in Section S8.

S6. Kinetic Equation for Resistance Transient for HSGC Layer Transfer

Using Langmuir adsorption-desorption kinetics the response and recovery of a sensor resistance transient can be written as:

$$R(t)_{response} = a + b.[1 - e^{-\frac{t}{c}}]$$

$$R(t)_{recovery} = b + d.e^{-\frac{t}{e}}$$

Here variable t is the measurement time and other parameters are respective adsorption and desorption parameters. Now for our case, specific gas molecule enters a specific layer, react with sensor (response) and leaves (recovery) and provides unique time instant (t_{max}) where resistance reaches maximum. To express mathematically the entire phenomena, the entire resistance transients should be the superposition of all and can be written as:

$$R(t)_{total} = a + b.[1 - e^{-\frac{t}{c}}] + b + d.\, e^{-\frac{t}{e}}$$

At $t = t_{max}$, $dR_{total}/dt = 0$

After solving this, we get, $t_{max} = \frac{c.e}{c-e}.\ln\left(\frac{dc}{eb}\right)$

After putting the t_{max} value we get, $R(t)_{total} = a + b.\left(1 - e^{-\frac{t}{c}}\right) + \frac{e}{c}.\, e^{t_{max}\frac{c-e}{c.e} - \frac{t}{e}}$

Now this t_{max} is the features of each VOC peaks in continuous chromatogram where each VOC come in different time and create separate t_{max} for each. Thus, the resultant equation of n number of VOC, is the superposition of each component.

$$R(t)_{total_{mix}} = a + \left[b_1.\left(1 - e^{-\frac{t}{c_1}}\right) + \frac{e_1}{c_1}.\, e^{t_{\max\_1}\frac{c_1-e_1}{c_1e_1} - \frac{t}{e_1}}\right] + \left[b_2.\left(1 - e^{-\frac{t}{c_2}}\right) + \frac{e_2}{c_2}.\, e^{t_{\max\_2}\frac{c_2-e_2}{c_1e_1} - \frac{t}{e_2}}\right] + \cdots \left[b_n.\left(1 - e^{-\frac{t}{c_n}}\right) + \frac{e_n}{c_n}.\, e^{t_{\max\_n}\frac{c_n-e_n}{c_ne_n} - \frac{t}{e_n}}\right] \quad \text{(S2)}$$

S7. VOCs Used for the Sensing Analysis

Table S3 | The list of used VOCs in mix VOC study.

**VOC type**	**Name of the VOC**
**Alcohols**	Methanol (VOC 1), Ethanol (VOC 2), Propanol (VOC 5), Butanol (VOC 7), Pentanol (VOC 12)
**Aldehydes**	Hexaldehyde (VOC 15), Heptaldehyde (VOC 18)
**Ketones**	Acetone (VOC 3), 2-butanone (VOC 6) Hexanone (VOC 14)
**Organic acid**	Acetic acid (VOC 4), Heptanoic acid (VOC 22)
**Hydrocarbon**	Benzene (VOC 8), cyclohexane (VOC 9), di-chloromethane (VOC 10), Hexane (VOC 11), Toluene (VOC 13), octane (VOC 19), xylene (VOC 16), ethylbenzene (VOC 17), 1,24- TMB (VOC 20), Nonane (VOC 21), Decane (VOC 23), dichlorbenzene (VOC 24)

***MVOC**= Mix VOC of all *e.g.*, MVOC_1 is the mixing ratio of methanol: other type of VOCs in 10 :1 molar ratio and MVOC_12 is the mixing ratio of pentanol: other type of VOCs in 10 :1 molar ratio.

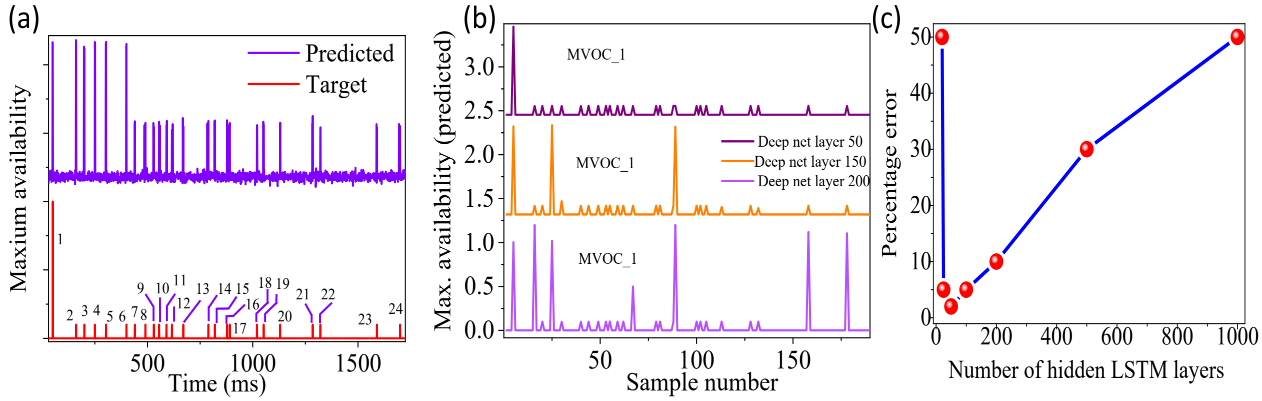

Figure S9. Optimization of deep net system. **a)** Prediction from uncompressed data set, VOC number is mentioned in Table S3. **b)** Prediction from wavelet based compressed data as input for various hidden layers (50-200). 50 hidden layer provides more than 95% accuracy. **c)** The optimization of hidden layer number from minimum error (or maximum accuracy) in prediction.

S8. Deep Learning Interface for Prediction of Structural/Chiral Chromatogram

Deep neural network architecture is constructed by MATLAB program with a typical sequence input layer which is fed from the HSGC derived time space resolved data (layer 2). This specific time-resistance sequence is further processed to LSTM layer and fully connected layers. Then finally a regression layer is added in the end to predict the chromatogram for continuous prediction. Due to large data set, we have used wavelet-based signal processing (with Haar level 3 decomposition) to compress the time sequence data (N) from the approximated coefficients values (N/2) and then used for deep neural network's sequence input layer. For the enantiomers mix state, we have used image processing by synthetically constructed input image from layer 2 HSGC data and fed to the self-learning architecture of deep net layers which has the following consecutive layers, such as, image-input layer, convolution2dLayer, reluLayer, maxPooling2dLayer, fully connected layer, classification layers. Before the training each image input sequence has been resized with same dimension. Deep net system automatically samples the features from image by itself and used for learning to classify the various mixed enantiomers and isomers. Figure S9a shows typical predicted chromatogram for MVOC-1 (methanol: other VOC type~10:1) using uncompressed data set from layer 2 HSGC. The predicted result does not match properly with the target ratio and could be due to large time stamp and data information and need to optimize the hidden layer information with longer training time. To solve this, HSGC derived input large data set is further compressed with wavelet transformation without losing important information and then used for deep net input as described above. Figure S9b shows the improved predicted chromatogram for various hidden deep net layers (50-200). Here 50 hidden layer shows the lowest error (see Figure S9c) for best prediction of the chromatogram and therefore used for prediction of other mixed vapors.

S9. Calculation of Chiral/Helical Cellulose and Chiral Enantiomer Interaction

After relaxing the geometry of host (cellulose) and target (enantiomer), docking simulation (generally followed for protein-ligand binding calculation) was done for finding lowest energy position to evaluate molecular dynamic simulation.[10] Typical boundary of the simulation box was set as 45x45x45 Grid size with resolution 0.4 Å in a flexible mode. Maximum number of host guest pose was set to 5000 to find the suitable position for binding. Cellulose has chiral center, and its natural helical structure provides distinct binding energy difference of each complex. The optimized geometry is shown in Figure S10 for (k) chiral cellulose-S(+)-butanol and (l) chiral cellulose- R(-)- butanol in the article.

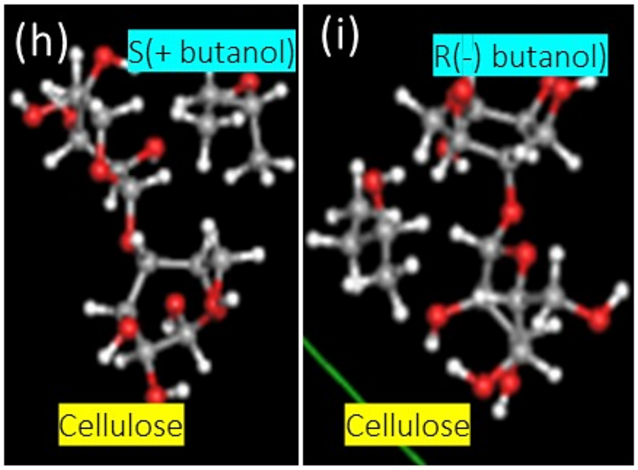

Figure S10. Optimized structure of chiral cellulose phase with S(+)butanol and R(-) butanol respectively using molecular dynamic simulation.

S10. DFT of 4-mercaptobenzoic acid (MBZA)-FDrGO Interaction with S(+)/ R(-)- Butanol

Gaussian 16 software was used to optimize the molecular structure as well as the HOMO and LUMO energy of FDrGO(MBZA) as well as their complexes R- and S-2-butanol molecules using a DFT method with Austin-Frisch-Petersson functional with dispersion (APFD) and the basis set 6-31G(d). APFD, including treatments of dispersion effects, represents the best trade-off between accuracy and computational cost for a relatively large system.

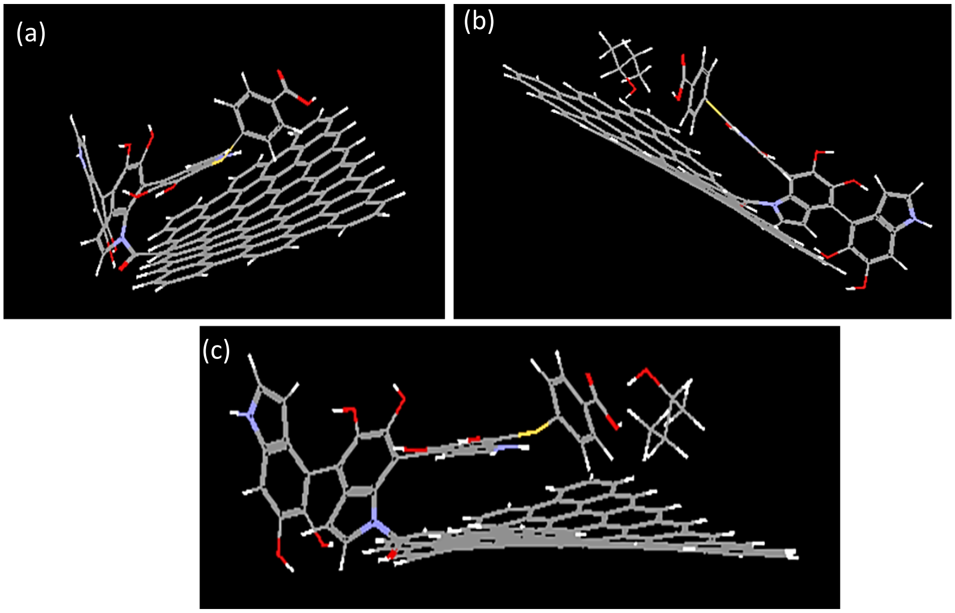

Figure S11. Optimized structure of sensor-target**.** DFT calculated optimized structure for **a)** FDrGO(MBZA), where MBZA=4 mercaptobenzoic acid, **b)** FDrGO(MBZA)-S(+) butanol, and **c)** FDrGO(MBZA)-R(-) butanol.

Table S4 Calculated HOMO-LUMO parameters from DFT analyses

	FDrGO(MBZA)	FDrGO(MBZA)-S(+) butanol	FDrGO(MBZA)-R(-) butanol
**HOMO**	-3.815	-3.773	-3.81
**LUMO**	-4.097	-4.05014	-4.085
**HOMO-LUMO gap**	0.281	0.276	0.273

The optimized structure for DFT calculation is shown for (a) FDrGO(MBZA), where MBZA=4 mercaptobenzoic acid as ligand, (b) FDrGO(MBZA)-S(+) butanol and (C) FDrGO(MBZA)-R(-) butanol complex Gibbs energy difference (ΔG) of S(+) butanol-host and R(-) butanol-host complex is -3332.76 and -6.34 kJ/mol, respectively. Calculated HOMO-LUMO gap of host-guest complex (for S-host and R-host) was calculated as 0.273 and 0.276 eV, respectively, and is lower than host (~0.281 eV) for both cases. Mullikan charge analyses show that butanol fragment of host-guest complex is positive for both case (0.0117 and 0.0137) and signifies that the butanol fragment for each enantiomer case donates the charge to the host sensor. HOMO-LUMO analyses also provided the same findings. Although these analyses could explain the interaction strength of each type of complex, no further conclusion could be drawn for the reason of opposite direction change of each enantiomer type. To explain further we have considered the spin polarization effect of the modified band structure of graphene due to presence of doping or defect.[11-12] In general, graphene is diamagnetic. However, it shows magnetic behavior when some foreign atom or defect is present in C-C hexagonal lattice.[11-12] Due to doping of other element (*e.g.*, H_2, N_2, F) time reversal symmetry breaks and due to orbital overlap of p electron, the local crystal structure exhibit finite magnetic moment. This make graphene magnetic, which is highly desirable for spintronics and chiral recognition.[11-12] In general, during the chiral based charge transfer, the interaction is also accompanied by spin injection which is completely opposite to each enantiomer type (see the schematic in Figure 3g in article). This phenomenon is commonly termed as chiral induced spin selectivity (CISS).[13-14] Using this unique spin injection, the surface become spin polarized (*i.e.*, majority spin direction is either up or down). Due to the specific spin population, energy band of graphene structure modulates (increased or decreased). This band splitting phenomena is also predicted by quantum mechanical DFT when graphitic structure is doped with various nitrogen-based ligand.[15] In our case, during the reduction of GO and polymerization reaction of polydopamine the oxygen containing group from C-C lattice is removed and N based compound from polydopamine chain is impregnated in C-C lattice (see reaction scheme at Figure S2). The presence of N_2 adatom breaks time and space reversal symmetry and possess finite magnetic moment and open the Dirac band gap and becomes n-type (see DFT analysis in Figure S12). In Figure S12, DFT analyses for two typical extreme case (considering all the spin configuration in N site is either all up or all down) and calculated band gap difference between these two cases shows that energy difference is indeed different in magnitude. This distinct band modulation (increase or decrease of band gap) due to specific spin population and polarization on the surface exhibits opposite directional resistance change (increase/decrease of resistance, see Figure 3h) for different enantiomer type using CISS mechanism. This result shows a high potential for the next generation spintronics, spin-based logic and spin-based memory/magnet free storage device applications. To further investigate about the specific spin influence during chiral exposure, magneto resistance measurement has been done and shown in Section S12.

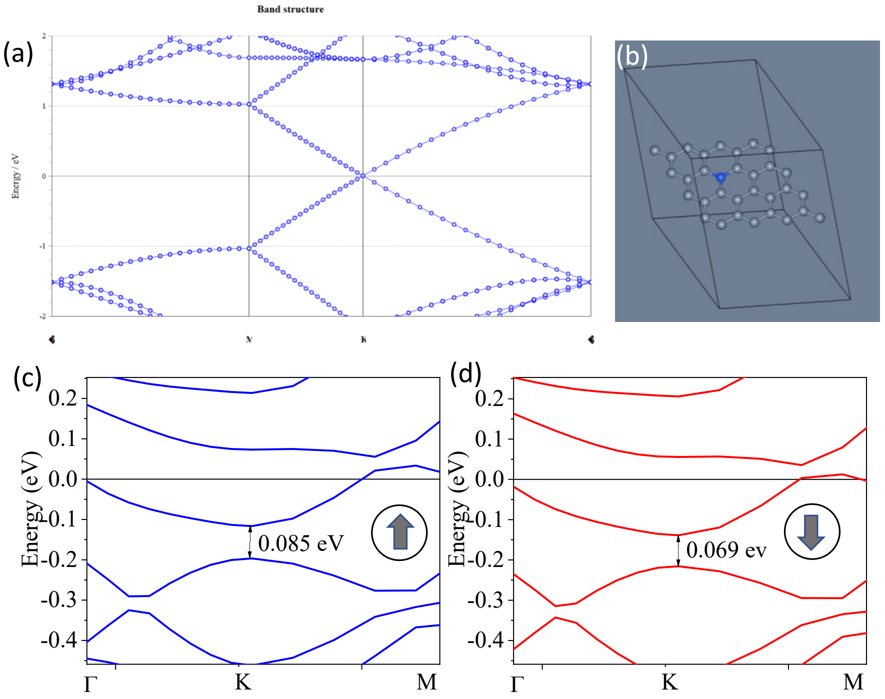

Figure S12. DFT study of spin influence in band gap. Calculated band structure of a) pristine graphene (Dirac cone at K point), b) Schematic of the N doped Graphene lattice, and its influence in band structure for c) spin up and d) spin down state.

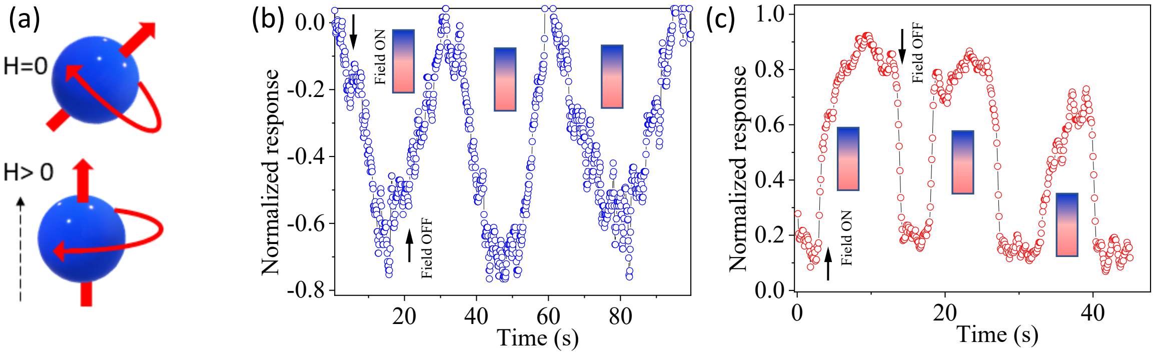

Figure S13. Magnetic measurement (MR) for determining spin influence. a) magnetic influence on spin for constant chiral exposure of b) S(+)-butanol and c) R(-)-butanol.

S11. Spin Influence from N-Doped Graphene on Band Gap

A spin- polarization calculation is presented using DFT for two typical extreme cases (considering the spin configuration in the N site as either <↑| or <↓|). The band gap difference in these two cases shows that the energy difference is indeed significant (Figure S12). This distinct band modulation (increase/decrease) due to a specific spin population and polarization on the surface causes opposite directional resistance changes. Although this is a very crude model, it can provide guidance about the influence of spin on enantiomer detection (see local magnetoresistance (MR) measurement in constant chiral flow, which shows opposite changes for each type (S(+) and R(-)), in Figure S13). This favors the idea of specific spin influences in chiral recognition[27-30]. We expect that the present observation will motivate further studies of detailed mechanisms and their application in chiral spintronics and topological quantum engineering of molecular graphenoid fields.

Ab-initio quantum DFT analysis of for electronic structure and band gap calculations is done for iterative solution of Kohn–Sham equation in a plane-wave set with the ultrasoft pseudopotentials[16]. Here Perdew–Burke–Ernzerhof (PBE) exchange–correlation (XC) functional of the generalized gradient approximation (GGA) is used. The plane-wave cutoff for wave function was set at 597 eV. The graphene sheets are separated by ~ 20 Å along the perpendicular direction to avoid interlayer influence. The Brillouin zone is sampled with Monkhorst–Pack scheme with and 7 7 1 k-mesh in gaussian smearing condition. The spin polarization was performed by replacing one carbon atom with N by setting the 100% spin up/down configuration at N site.

S12. Analyses of Data from Malignant/Non-Malignant Tissues

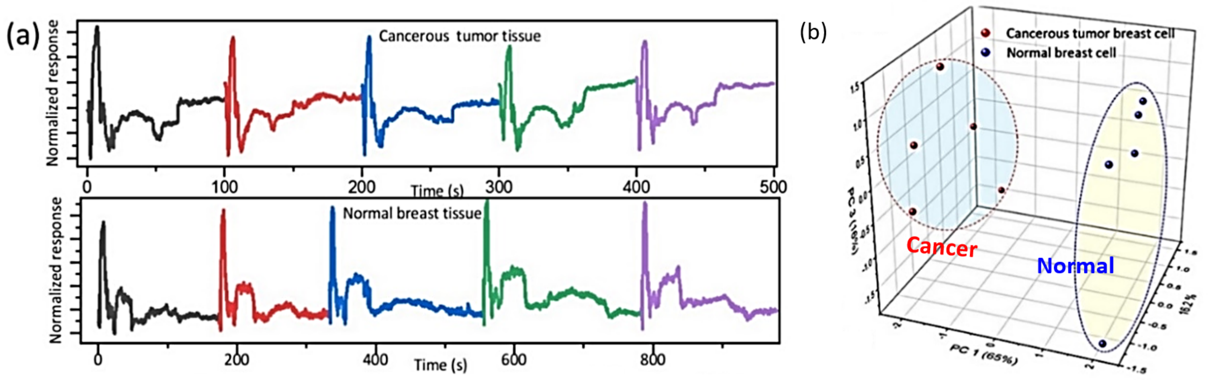

Figure S14. Reproducibility and statistical analyses of data from malignant/non-malignant tissues. a) Typical reproducibility from multiple cycles shows the consistent separation of VOC components characteristic of malignant breast tumors and healthy breast tissues from multiple clinical samples. b) PCA analyses to separate cancer from normal breast tissues from spectrogram information of HSGC sensing devices.

S13. Molecular Spectral Chromatogram from Skin-Emitted VOCs

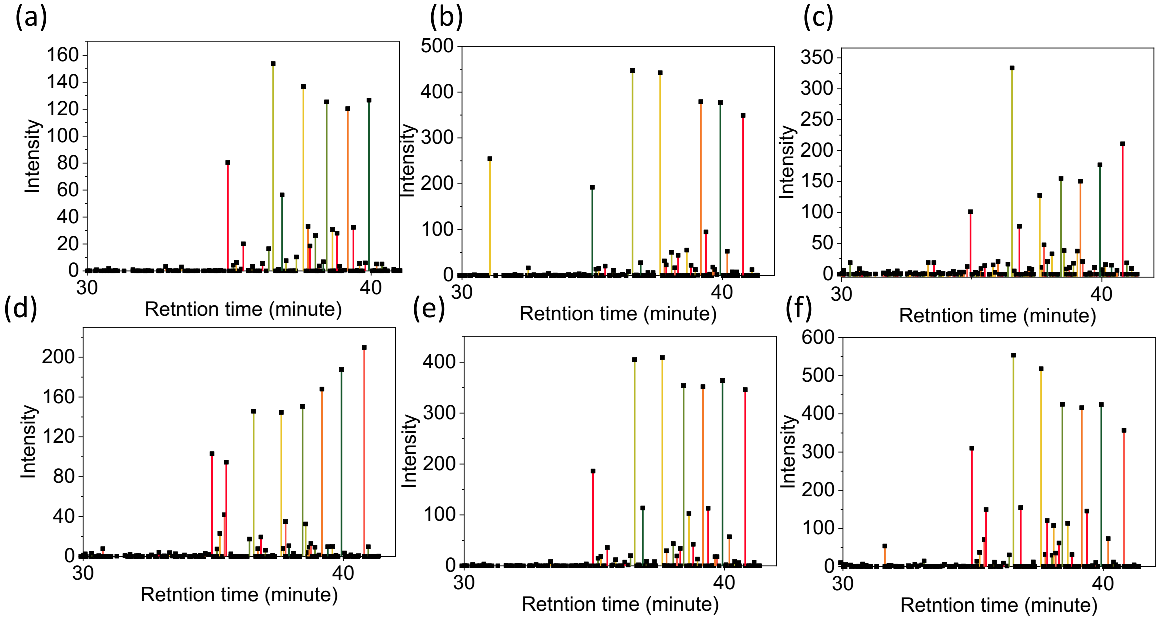

Figure S15. The GC-MS results of dietary intake from skin extracted samples for (a-f) dietary influences such as, gluten, caffeine, dairy, fatty meal, cigarette smoke, and sugary product respectively (see list of VOCs in Table S6).

Table S5 | The list of VOCs from GC-MS result of various dietary effect extracted from PDMS strip.

VOC label	VOC names
1	Isocyanic acid
2	1,2-Benzenedicarboxylic acid, monobutyl ester
3	Thiochalcone
4	3-(1-Hydroxyhexyl)phenol
5	1-Dodecanol
6	1,6-Dichloro-2,5-dimethylhexane
7	Butane
8	Hexadecane
9	1-Butyl-1H-1,2,4-triazole
10	Benzoic acid
11	Hexadecanoic acid
12	Bicyclo[3.1.1]heptan-3-one
13	Heptadecanoic acid
14	Ethanol
15	Sulfurous acid
16	Dibutyl phthalate
17	Butyl myristate
18	2,6-Dimethyl-6-phenylthio-9-(1-methyl-ethenyl)decen-7-one
19	(4-Fluorobenzoyl)(tert-butyl)dimethylsilane
20	Nonane,
21	1-Butanol
22	Dodecyl octyl ether
23	3-ethenyl-3-methyl-4-pentenal

24	2,2-dimethyl-1-nitropropane
25	Sulfurous acid
26	(E)-2-(2H(1)-4-Methoxyphenylethene
27	p-Tolylpentamethyl-disiloxane
28	1,3-Diphenyl-1-trimethylsilyloxy-1-pentene
29	1-(2-(4-methoxyphenyl)ethenylcarbonyloxyl)3-methylbutane
30	Methylsilyl Formate
31	(+-)-3,5-Dimethyl-3,6-dihydro-2H-1,
32	Cyclododecanemethanol
33	Hexadecane
34	1-Octadecenal,
35	Methoxycyclopentaneacetonitrile
36	(S)-4-Iodo-1,2-epoxybutane
37	6-methyloctahydrocoumarin
38	Hexadecanoic acid
39	2-Ethylhexyl trans-4-methoxycinnamate
40	(t-Butyl)-4,5-bis(trifluoromethyl)-1,3,2-dithiazol
41	5-Methylsalicylic acid
42	N,N'-dimethylbenzamidine
43	1-Decanol
44	3-(1-Hydroxyhexyl)phenol
45	Diallyldivinylsilane
46	Nopinone
47	(Z,Z)-1,14-dibromo-2,12-tetradacadiene
48	propionic acid
49	1-Phenyl-4-(2'-bromophenyl)-1-butyne
50	pentadecane
51	3(R,S)-hydroxy-8(S)-12-dimethyl-1,5(E)
52	4-Hydroxybenzyl alcohol
53	1,5,9-Undecatriene
54	alpha.-Dimethoxymesitylene
55	4-[[6-(Ethoxycarbonyl)-trans-5-hexenyl]oxy]-6-methyl-2-pyrone
56	2,5-Diethoxyaniline
57	[(t-butyl)dimethylsilyl] propionate
58	Thiophen-2-methylamine
59	Pentadecane
60	Hexamethyl-Cyclotrisiloxane
61	9-trans-9,10-epoxyretinoic acid
62	pentadecane
63	2,6-Dideutero-pyridine
64	2,2,2-trichloroethyl ester ethanoic acid
65	9-trans-9,10-epoxyretinoic
66	Benzeneacetic acid,
67	acetonyl decyl ether
68	Ethanoic acid 2,2,2-trichloroethyl ester
69	3-tetradecenyl-1,2-bis(trimethylsilyl)glyceryl ether
70	Dimethoxy-methane
71	.beta.,D-Xylopyranose Tetrabenzoate
72	Cyclotetrasiloxane
73	Cpd 53: 3-azatricyclo[3.1.1.1(2,4)]octane
74	Cyano(3-cyclohexenyl)methane
75	2-(2-ethoxyethoxy)-Ethanol,
76	Octanal

77<br>78<br>79<br>80<br>81<br>82<br>83<br>84<br>85<br>86<br>87<br>88<br>89	1-Hexanol<br>2-Pyrrolidinone<br>Cyclotrisiloxane<br>1-Cyclohexyl-2-nitro-1,3-propanediol<br>Methylamine,<br>hexane-2,4-diol<br>2-Pyrrolidinone<br>Nonanal<br>Benzeneacetic acid<br>Ethanol<br>3-Methylheptyl acetate<br>Benzoic acid, butyl ester<br>1-Hexene<br>2-(2-butoxyethoxy)- Ethanol<br>1-Decanol<br>2-methoxy[1]benzothieno[2,3-c]quinolin-6(5H)-one<br>(2S)-2-methylbutanal<br>Decanal<br>hept-6-en-1-yl)-tert-butyl-ether<br>4-formylmethyl-2-propyl-1,3-dioxolane<br>3(R)-fluoro-4-methyl-1,4-pentanediol<br>Hexaoxacycloeicosane<br>Tridecanoic acid

References:

1. L. Q. Xu, W. J. Yang, K.G. Neoh, E.T. Kang, and G. D. Fu, Dopamine-Induced Reduction and Functionalization of Graphene Oxide Nanosheets. *Macromolecules* **43**, 8336–8339 (2010). DOI:10.1021/ma101526k.
2. G. Hu, J. Kang, L. W. T. Ng, X. Zhu, R. C. T. Howe, C. G. Jones, M. C. Hersam, T. Hasan, Functional inks and printing of two-dimensional materials. *Chem. Soc. Rev*. **47**, 3265--3300 (2018). DOI: doi.org/10.1039/C8CS00084K.
3. J. H. Waite, M. L. Tanzer, Polyphenolic Substance of Mytilus edulis: Novel Adhesive Containing L-Dopa and Hydroxyproline. *Science* **212**, 1038–1040 (1981). DOI: 10.1126/science.212.4498.1038.
4. R. F. Hossain, I. G. Deaguero, T. Boland and A. B. Kaul, Biocompatible, large-format, inkjet printed heterostructure MoS_2-graphene photodetectors on conformable substrates. *NPJ 2D Mater. Appl.* **1**, 28 (2017). DOI: 10.1038/s41699-017-0034-2.
5. S. Santra, G. Hu, R. C. T. Howe, A. De Luca, S. Z. Ali, F. Udrea, J. W. Gardner, S. K. Ray, P. K. Guha and T. Hasan, CMOS integration of inkjet-printed graphene for humidity sensing. *Sci. Rep*., **5**, 17374 (2015). DOI: 10.1038/srep17374 (2015).
6. C. Sriprachuabwong, C. Karuwan, A. Wisitsorrat, D. Phokharatkul, T. Lomas, P. Sritongkham and A. Tuantranont, Inkjet-printed graphene-PEDOT:PSS modified screen printed carbon electrode for biochemical sensing. *J. Mater. Chem*., **22**, 5478 (2012). DOI: doi.org/10.1039/C2JM14005E.
7. K. Arapov, R. Abbel, G. de With and H. Friedrich, Inkjet printing of graphene. *Faraday Discuss*. **173**, 323–336 (2014). DOI: 10.1039/C4FD00067F
8. E. B. Secor, T. Z. Gao, A. E. Islam, R. Rao, S. G. Wallace, J. Zhu, K. W. Putz, B. Maruyama and M. C. Hersam, Enhanced Conductivity, Adhesion, and Environmental Stability of Printed Graphene Inks with Nitrocellulose. *Chem. Mater*., **29**, 2332–2340 (2017). DOI: 10.1021/acs.chemmater.7b00029.
9. M. Parente, M. van Helvert, R. F Hamans, R. Verbroekken, R. Sinha, A. Bieberle-Hütter, and A. Baldi, Simple and Fast High-Yield Synthesis of Silver Nanowires. *Nano Lett*, **20**, 8, 5759–5764 (2020). DOI: 10.1021/acs.nanolett.0c01565.
10. M.A. Thompson, Molecular Docking Using ArgusLab (2004).
11. R. Babar and M. Kabir, Ferromagnetism in nitrogen-doped graphene, *Physical review. B* **99**, 115442 (2019). DOI: 10.1103/PhysRevB.99.115442.
12. W. Han, R. K. Kawakami, M. Gmitra and J. Fabian, Graphene spintronics. *Nat. Nanotech.* **9**, 794–807 (2014). DOI: 10.1038/nnano.2014.214.
13. Ron Naaman, Yossi Paltiel and David H. Waldeck, Chiral molecules and the electron spin. *Nat. Rev. Chem*. **3**, 250–260 (2019). DOI: doi.org/10.1038/s41570-019-0087-1.
14. S. Yang, R. Naaman, Y. Paltiel and S. S. P. Parkin, Chiral spintronics. *Nat. Rev. Phys*. **3**, 328–343 (2021). DOI: 10.1038/s42254-021-00302-9.
15. F. López-Urías, J. L. Fajardo-Díaz, A. J. Cortés-López, C. L. Rodríguez-Corvera, L. E. Jiménez-Ramírez, E. Muñoz-Sandoval, Spin-dependent bandgap driven by nitrogen and oxygen functional groups in zigzag graphene nanoribbons. *Appl. Surf. Sc*. **521** 146435 (2020). DOI: 10.1016/j.apsusc.2020.146435.
16. P. Giannozzi, S. Baroni, N. Bonini, M. Calandra, R. Car, C. Cavazzoni, D. Ceresoli, G. L. Chiarotti, M. Cococcioni, I. Dabo, A. Dal Corso, S. Fabris, G. Fratesi, S. de Gironcoli, R. Gebauer, U. Gerstmann, C. Gougoussis, A. Kokalj, M. Lazzeri, L. Martin-Samos, N. Marzari, F. Mauri, R. Mazzarello, S. Paolini, A. Pasquarello, L. Paulatto, C. Sbraccia, S. Scandolo, G. Sclauzero, A. P. Seitsonen, A. Smogunov, P. Umari, R. M. Wentzcovitch, QUANTUM ESPRESSO: a modular and open-source software project for quantum simulations of materials. *J. Phys.: Condens.Matter* **21**, 395502 (2009). DOI: 10.1088/0953-8984/21/39/395502.
